# *Klebsiella pneumoniae* K2 capsular polysaccharide degradation by a bacteriophage depolymerase does not require trimer formation

**DOI:** 10.1128/mbio.03519-23

**Published:** 2024-02-13

**Authors:** Ting-Juan Ye, Kit-Man Fung, I-Ming Lee, Tzu-Ping Ko, Chia-Yi Lin, Chia-Ling Wong, I-Fan Tu, Tzu-Yin Huang, Feng-Ling Yang, Yu-Pei Chang, Jin-Town Wang, Tzu-Lung Lin, Kai-Fa Huang, Shih-Hsiung Wu

**Affiliations:** 1 Institute of Biological Chemistry, Academia Sinica, Taipei, Taiwan; 2 Department of Marine Biotechnology and Resources, National Sun Yat-sen University, Kaohsiung, Taiwan; 3 Department of Microbiology, National Taiwan University College of Medicine, Taipei, Taiwan; 4 Department of Internal Medicine, National Taiwan University Hospital, Taipei, Taiwan; 5 Institute of Biochemical Sciences, National Taiwan University, Taipei, Taiwan; University of Pittsburgh, USA

**Keywords:** *Klebsiella pneumonia*, bacteriophage, tailspike protein, *O*-acetylation, intersubunit carbohydrate-binding site

## Abstract

K2-capsular *Klebsiella pneumoniae* is a hypervirulent pathogen that causes fatal infections. Here, we describe a phage tailspike protein, named K2-2, that specifically depolymerizes the K2 capsular polysaccharide (CPS) of *K. pneumoniae* into tetrasaccharide repeating units. Nearly half of the products contained *O*-acetylation, which was thought crucial to the immunogenicity of CPS. The product-bound structures of this trimeric enzyme revealed intersubunit carbohydrate-binding grooves, each accommodating three tetrasaccharide units of K2 CPS. The catalytic residues and the key interactions responsible for K2 CPS recognition were identified and verified by site-directed mutagenesis. Further biophysical and functional characterization, along with the structure of a tetrameric form of K2-2, demonstrated that the formation of intersubunit catalytic center does not require trimerization, which could be nearly completely disrupted by a single-residue mutation in the C-terminal domain. Our findings regarding the assembly and catalysis of K2-2 provide cues for the development of glycoconjugate vaccines against *K. pneumoniae* infection.

**IMPORTANCE:**

Generating fragments of capsular polysaccharides from pathogenic bacteria with crucial antigenic determinants for vaccine development continues to pose challenges. The significance of the C-terminal region of phage tailspike protein (TSP) in relation to its folding and trimer formation remains largely unexplored. The polysaccharide depolymerase described here demonstrates the ability to depolymerize the K2 CPS of *K. pneumoniae* into tetrasaccharide fragments while retaining the vital *O*-acetylation modification crucial for immunogenicity. By carefully characterizing the enzyme, elucidating its three-dimensional structures, conducting site-directed mutagenesis, and assessing the antimicrobial efficacy of the mutant enzymes against K2 *K. pneumoniae*, we offer valuable insights into the mechanism by which this enzyme recognizes and depolymerizes the K2 CPS. Our findings, particularly the discovery that trimer formation is not required for depolymerizing activity, challenge the current understanding of trimer-dependent TSP activity and highlight the catalytic mechanism of the TSP with an intersubunit catalytic center.

## INTRODUCTION

*Klebsiella pneumoniae* is a gram-negative, non-motile, encapsulated bacterium that belongs to the *Enterobacteriaceae* family. In the past 30 years, *K. pneumoniae* has become hypervirulent and resistant to antibiotics by acquiring genetic traits (1). Hypervirulent *K. pneumoniae* (hvKp) is responsible for pyogenic liver abscesses, pneumonia, urinary tract infections, surgical site infections, and intra-abdominal infections (2, 3). It has become one of the nosocomial pathogens in North America and Europe and is especially common in Asia among immunocompromised patients (2, 4, 5). The virulence factors of *K. pneumoniae* include siderophores, pili, and capsular polysaccharides (CPS) (6). The CPS is produced by proteins encoded in the capsular polysaccharide synthesis (*cps*) gene locus and surrounds the outer layer of the cell surface via a lipid anchor (7, 8). CPS can be linear or branched and is usually comprised of repeating units of oligosaccharide with non-carbohydrate substitutes via glycosidic linkages (9, 10). *K. pneumoniae* employs CPS as a crucial defense mechanism to protect itself against the host’s immune system during infection. This outer layer acts as a shield, effectively thwarting the early immune responses initiated by the host’s immune cells, such as neutrophils and macrophages. Furthermore, CPS provides an additional safeguard by preventing damage caused by antimicrobial peptides and complement-mediated lysis (1, 11, 12). Over 79 capsular serotypes have been identified in *K. pneumoniae*, with K1 and K2 being the most commonly isolated serotypes, accounting for approximately 70% of hvKp isolations (13, 14).

As an alternative to antibiotic treatment, vaccines targeting bacterial CPS have demonstrated efficacy in combatting infections by pathogenic bacteria, including *Streptococcus pneumoniae*, *Neisseria meningitidis*, and *Haemophilus influenzae* (15, 16). While whole CPS isolates from pathogenic bacteria induced relatively poor and T cell-independent immune responses (17), glycoconjugate vaccines created by conjugating CPS fragments to a carrier protein such as CRM197 were found to elicit stronger and long-lasting immune responses (18–20). Furthermore, bacteriophages can depolymerize the CPS of their host bacteria through the action of their polysaccharide depolymerases (21). The resulting digested products typically consist of oligosaccharides with repeating units, which preserve the immunogenicity of CPS. This underscores the therapeutic potential of bacteriophage-derived polysaccharide depolymerases and their resulting oligosaccharide products in the development of vaccines against *K. pneumoniae* infections (22–25).

Bacteriophages are viruses that specifically infect certain bacterial hosts. They can have tail spikes or tail fibers (or sometime both); tail spikes usually refer to more compact proteins that have some enzymatic activity, and tail fibers are longer and bind passively to their receptors without enzymatic activity. During the initial stage of infection, the tailspike proteins (TSPs) of bacteriophage recognize specific polysaccharides on the surface of the host cell and attach to them for adsorption (26, 27). TSPs, also known as polysaccharide depolymerases, have the function of binding and digesting CPS. The existence of TSPs was first reported in 1956 through the plaque surrounding halos experiment (28, 29). To better understand TSPs, the crystal structure of P22 TSP from *Salmonella* phage has been studied. P22 TSP forms a homotrimer, which is believed to be crucial for its catalytic activity, and it serves as a model for other TSPs (30–33). Since the discovery of P22, many polysaccharide depolymerase TSPs have been characterized (33–36). TSPs typically consist of three domains: an N-terminal particle-binding domain that connects to the tail structure or baseplate of the phage, a receptor-binding domain that extends from the homotrimeric structure and is responsible for host recognition and catalytic activity, and a C-terminal domain that plays a vital role in trimer formation, receptor binding, and intra-molecular chaperone functions (26, 27, 30, 31, 33). TSPs demonstrate stability under different temperature and pH conditions, as well as resistance to SDS or proteinases, which make them suitable for various biotechnical applications (21, 27).

In the present study, we characterize a phage-derived polysaccharide depolymerase specific to the K2 CPS of *K. pneumoniae*, named K2-2, whose amino-acid sequence is distinct from the recently reported K2 CPS depolymerase 1611E-K2-1 (23). The hydrolyzed K2 CPS products by K2-2 were also identified. The crystal structures of K2-2 in complex with the hydrolyzed CPS products were determined, revealing a trimeric architecture with intersubunit carbohydrate-binding grooves that accommodate three tetrasaccharide repeating units of K2 CPS. Combined with site-directed mutagenesis, the structures allowed for the identification of the catalytic residues as well as the key interaction between K2 CPS and K2-2 that is responsible for K2 CPS recognition by the enzyme. Further site-directed mutagenesis and biophysical studies also revealed the key interactions in the C-terminal domain of the enzyme that are crucial to its trimerization. In addition, we obtained a tetrameric form of this enzyme and determined its crystal structure, which demonstrated for the first time the formation of a functional intersubunit catalytic center without the need for trimerization.

## RESULTS AND DISCUSSION

### Characterization of recombinant K2-2 and its hydrolyzed K2 CPS products

To evaluate the host specificity of the bacteriophage encoding K2-2, an assessment of capsule degradation activity of this phage against 80 different capsular serotypes of *K. pneumoniae* was conducted. The results demonstrate that the phage is capable of degrading the CPS of K2 and K13 serotypes of *K. pneumoniae* (Table S1). The K13 CPS, however, was reported to share the same polysaccharide backbone as the K2 CPS (37).

**Fig 1:**
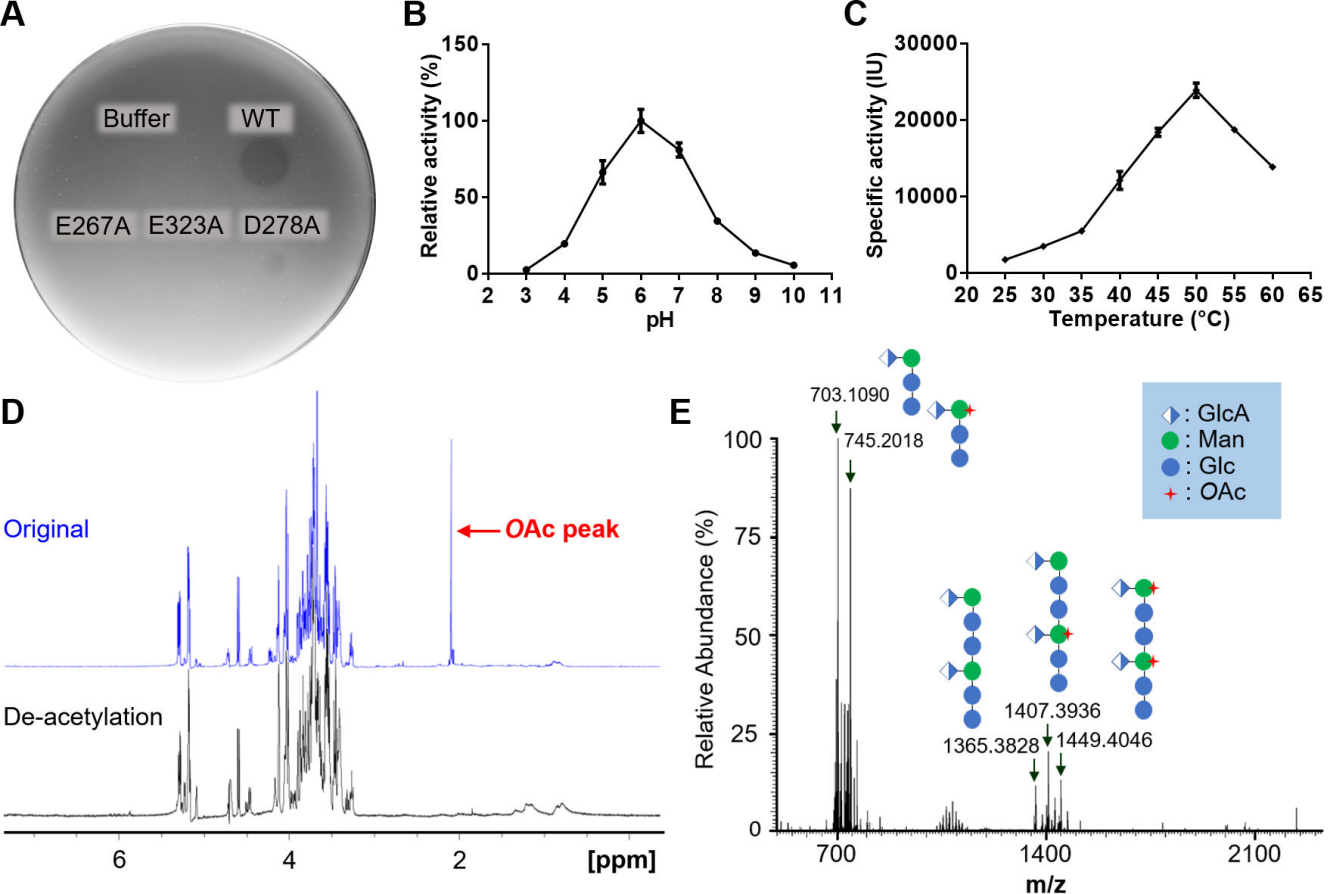
Characterization of recombinant K2-2 and its hydrolyzed products of *K. pneumoniae* K2 CPS. (**A**) Evaluation of capsule degradation activity of wild-type and mutant K2-2 against the K2 *K. pneumoniae* NTUH-A4528*ΔwbbO* by top agar assay. These mutations target the proposed catalytic residues, as described in the Section 2.3. (**B**) CPS hydrolyzing activity of K2-2 at various pH conditions. Data are shown as means ± SD from three replicates. (**C**) Specific enzyme activity of K2-2 at various temperatures under pH 6.0. Data are shown as means ± SD from three replicates. IU: international unit of enzyme activity, defined as μmole/min. (**D**) ^1^H NMR spectrum (500 MHz) of hydrolyzed K2 CPS products with (black) and without (blue) the pretreatment of deacetylation. The signal corresponding to the *O*-acetyl group is marked. (**E**) LC-ESI-MS analysis of hydrolyzed K2 CPS products. The molecular mass and *O*-acetylation of the products are further indicated.

The recombinant K2-2 with an N-terminal His-tag was overexpressed in *E. coli* cells and purified to homogeneity. The purified enzyme generated a translucent halo when spotted on agars overlaid with the K2 *K. pneumoniae* NTUH-A4528*ΔwbbO* (Fig. 1A), demonstrating its K2 CPS depolymerization activity. Further characterization of the enzyme indicated an optimal catalytic activity of 23,902 ± 939 IU at pH 6.0 and 50°C (Fig. 1B and C). As revealed by 1D (^1^H, ^13^C, and TOCSY) and 2D NMR (COSY, HSQC, HMBC, and NOESY), the hydrolyzed products of K2 CPS contained one or two repeating units of the tetrasaccharide  → 4)-[α-Glc*p*A-(1→3)]-β-Man*p*-(1→4)-α-Glc*p*-(1→3)-β-Glc*p*-(1→ (File S1). By analyzing the ^1^H NMR spectrum, we noticed that approximately 30%–50% of the products possessed *O*-acetylation (Fig. 1D), suggesting that this modification is not a prerequisite for K2 CPS degradation by the enzyme. Further analysis of the hydrolyzed products by LC–ESI–MS revealed the major species of one tetrasaccharide repeating unit with (*m*/*z* = 745) or without (*m*/z = 703) an *O*-acetylation, accompanied by the minor products corresponding to two repeating units with one (*m*/*z* = 1,407), two (*m*/*z* = 1449), or without (*m*/*z* = 1,365) the *O*-acetylation (Fig. 1E). Notably, the hydrolyzed products described here are quite different from the recently reported ones made by the K2 CPS depolymerase DpK2 (38), which produced 2 and 3 repeating units of the tetrasaccharide →3)-β-Glc*p*-(1→ 4)-[α-Glc*p*A-(1→3)]-β-Man*p*-(1→4)-α-Glc*p*-(1→. Given that *O*-acetylation on bacterial CPSs is deemed crucial for their immunogenicity, the oligosaccharide products made by K2-2 here hold promise for application in the development of glycoconjugate vaccines against K2 *K. pneumoniae* infection (39–41).

### K2-2 exhibits a trimeric superhelical structure

**Fig 2:**
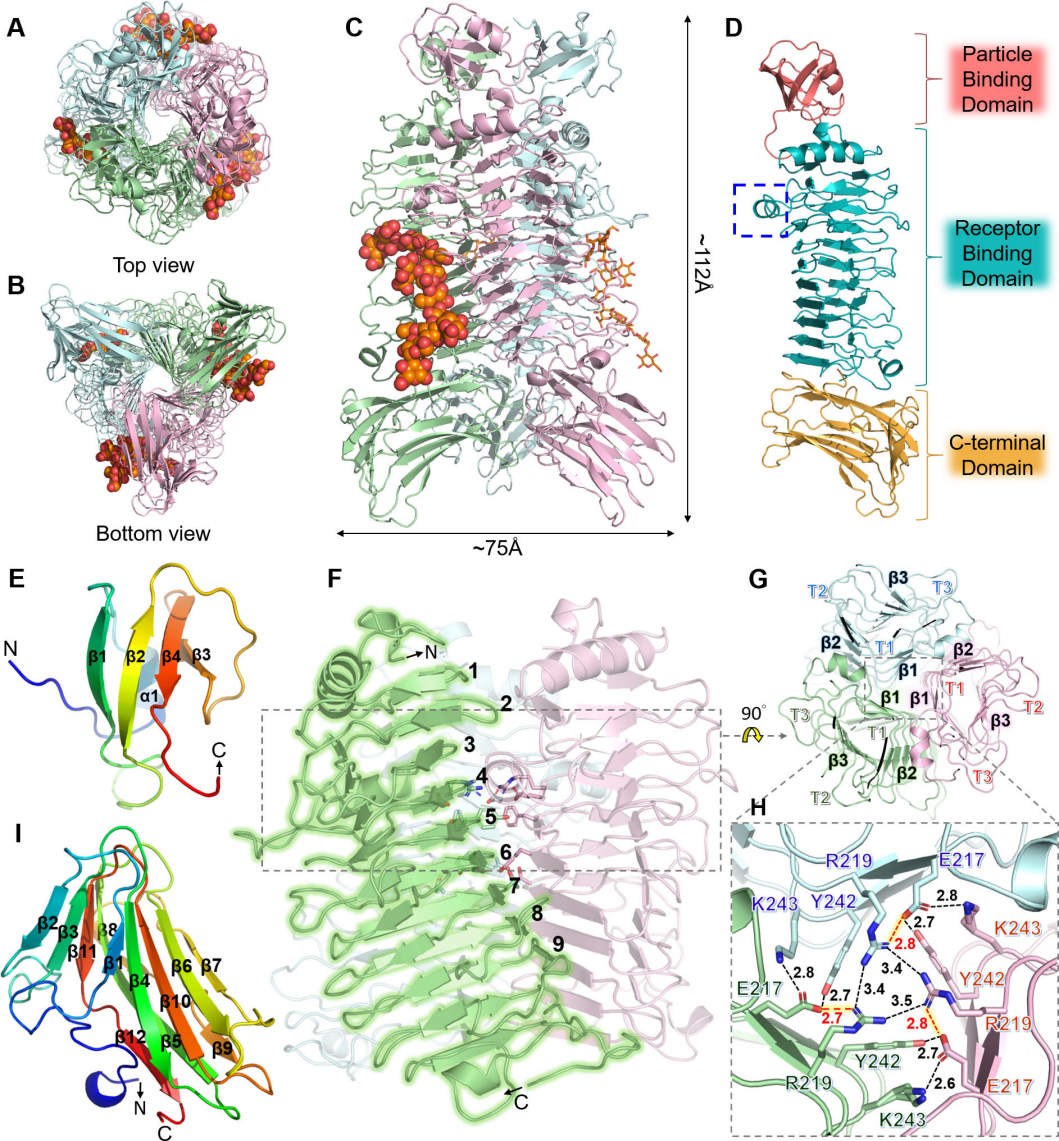
Crystal structure of K2-2. (**A**–**C**) Overall structure of K2-2 in top, bottom, and side view, respectively (PDB: 8IQ9). The homotrimeric structure is drawn as a three-colored ribbon, with the bound oligosaccharide products being shown as space-filling or stick models in orange. (**D**) Structure of individual subunit (PDB: 8IQ5). The three domains are further indicated. (**E**) A close-up view of the particle-binding domain in individual subunit. (**F**) A close-up view of the central receptor-binding domain (RBD). The residues involved in inter-subunit interactions are shown as stick model. (**G**) A cross-section in RBD of a trimer. The RBD in individual subunit folds into a β-helix of nine rungs, each rung being composed of three β-strands (β1, β2, and β3), separated by three turns (T1, T2, and T3). (**H**) The triangular interaction network in the trimer interface of RBD. The three pairs of Glu217 and Arg219, each from a different subunit, form three inter-chain hydrogen bonds (black dotted lines) and three intra-chain salt bridges (red dotted lines). (**I**) A close-up view of the C-terminal domain in individual subunit.

The recombinant K2-2 was crystallized in a monoclinic *P*2_1_ unit cell with the asymmetric unit containing two trimers of the enzyme, which were virtually identical. The crystal structure of K2-2 was solved at 1.38 Å resolution (Table S2). Later, a product-bound structure of the enzyme was determined in a distinct space group, as described in the following section, which showed an overall structure nearly identical to the free-form enzyme (Cα rmsd = 0.19 Å) (Fig. 2).

The structure of K2-2 exhibits a compact trimeric architecture with a length of ~112 Å and a diameter of ~75 Å in its widest region. Each monomer is organized into the putative N-terminal particle-binding domain, the receptor-binding domain, and the C-terminal domain (Fig. 2A through D). The particle-binding domain (residues 15–85) folds into a barrel-like structure consisting of a four-stranded β-sheet and a two-turn α-helix (Fig. 2E). To form a trimer, three equivalent barrels are associated with a dome-like structure (Fig. 2A), where three β-sheets face toward the dimer interface and three α-helices are solvent exposed.

The receptor-binding domain (residues 86–420) of each monomer folds into a right-handed β-helix composed of 9 complete rungs, with an N-terminal four-turn α-helix (residues 101–114) capping the lumen of the β-helix (Fig. 2F). Individual rung is organized into three strands (β1, β2, and β3) separated by three turns (T1, T2, and T3) (Fig. 2G). The central body of the β-helix displays a kidney-shaped cross-section with a well-formed hydrophobic core. Notably, a two-turn α-helix is inserted into the T3 of rung-2 and protrudes prominently from the border of the β-helix. In the trimer, three β-helices are packed laterally through β-sheets, forming a parallel left-handed superhelix with a hydrophilic channel at the center. The interior of the channel features a triangular interaction network consisting of three inter-chain hydrogen bonds and three intra-chain salt bridges made by three pairs of Glu217 and Arg219, each from a different subunit (Fig. 2H). Other inter-chain hydrogen bonds such as Glu217···Tyr242 and Glu217···Lys243 were observed as well (Fig. 2H; Fig. S1A). Additionally, the protruding α-helix interacts with an adjacent β-helix via hydrogen bonds to its β2 strands of rung-4 and rung-5 (Fig. S1B). The C-terminal domain (residues 421–577) of each monomer is organized into a β-sandwich topology, consisting of two antiparallel six-stranded β-sheets, plus an N-terminal one-turn α-helix (Fig. 2I). The three β-sandwiches in a trimer are then associated into a triangular prism (Fig. 2B).

### Product-bound structure reveals intersubunit carbohydrate-binding grooves that accommodate three repeating units of tetrasaccharide

**Fig 3:**
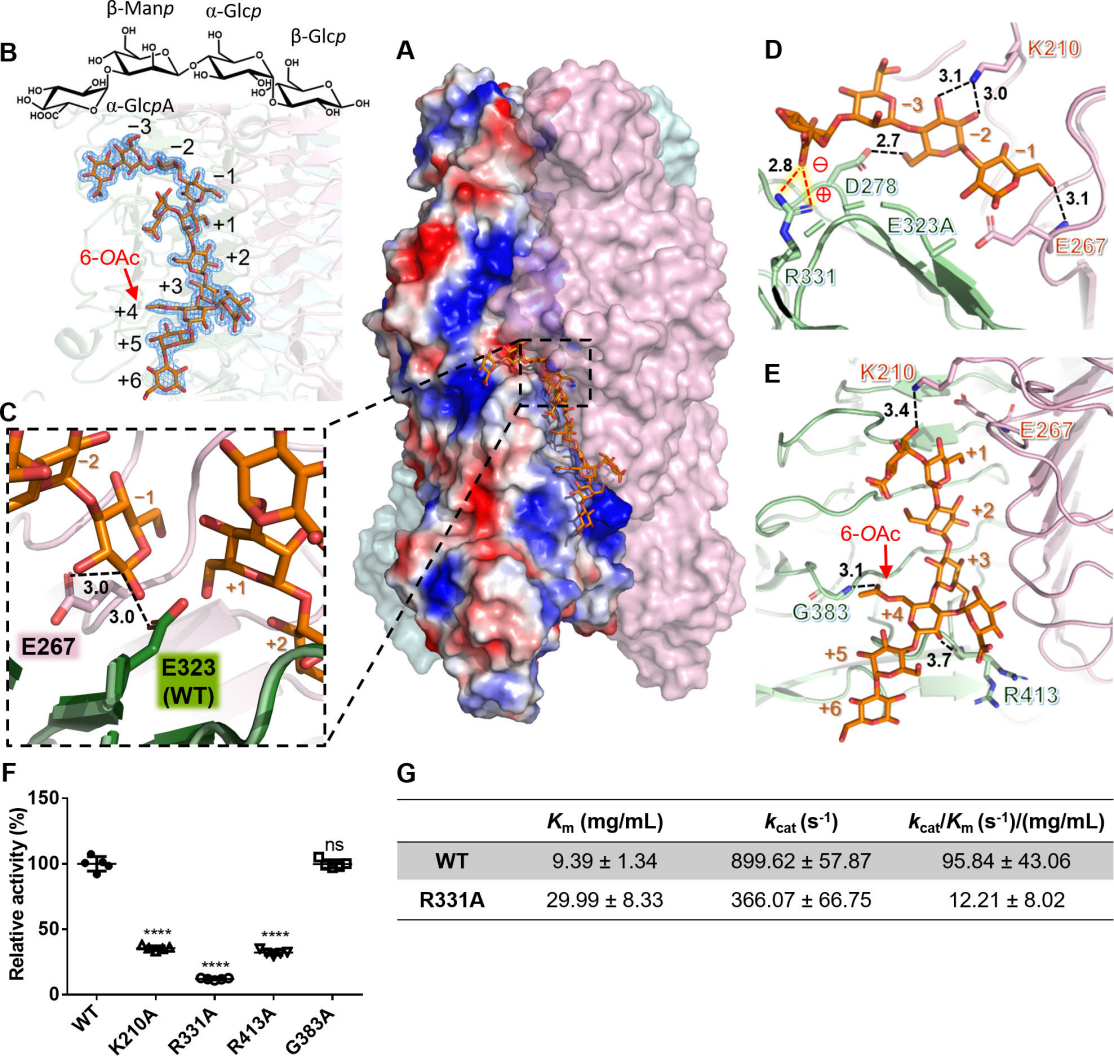
The intersubunit carbohydrate-binding sites bound with the hydrolyzed products of K2 CPS. (**A**) Surface representation of the structure of product-bound K2-2 (PDB: 8IQ9). The view is perpendicular to the trimer axis onto the intersubunit carbohydrate-binding groove with the N-terminal end on top. The bound oligosaccharide products are shown as stick model in orange. The surface charge potentials of a subunit are shown as well with blue and red colors representing the positive and negative charge, respectively. (**B**) A close-up view of the bound products. The 1*σ* 2*F*_o_*–F*_c_ omit map around one of the bound oligosaccharides is shown. The numbering of polysaccharide chain from −3 to +6 is based on the proposed hydrolysis site as described in the text. The *O*-acetylation at the +4 Man*p* is marked with a red arrow. The chemical structure corresponding to one tetrasaccharide repeating unit of K2 CPS is shown on top. (**C**) Proposed catalytic center. E323^WT^: Glu323 of wild-type K2-2, obtained by overlaying the E323A structure (pale green) with that of wild-type enzyme (forest). (**D**) A close-up view of the interaction of the bound one-repeating-unit oligosaccharide with K2-2. The salt bridge between the negatively charged carboxyl group of the oligosaccharide and the positively charged Arg331 of the enzyme is further indicated. (**E**) A close-up view of the interaction of the bound two-repeating-unit oligosaccharide with K2-2. The *O*-acetyl group at the C6-OH of +4 Man*p* is marked. (**F**) Comparison of the CPS hydrolyzing activity of K2-2 mutants K210A, R331A, R413A, and G383A with that of wild-type enzyme. The activities were measured under the optimal pH and temperature condition as described in the text. Data are shown as means ± SD from five replicates. ****P < 0.0001, calculated from one-way ANOVA. ns, no statistical significance. (**G**) Enzyme kinetic study of K2-2 and the mutant R331A.

Despite testing numerous soaking and co-crystallization conditions with the crude extract or hydrolyzed products of K2 CPS, we were unable to obtain a sugar-bound structure using the wild-type enzyme. Therefore, we decided to use inactive mutants of the enzyme for subsequent soaking and co-crystallization experiments. By analyzing the surface properties of the receptor-binding domain of the enzyme, we discovered a prominent negatively charged cavity located at the intersubunit interface, formed by the conserved carboxylate triad Asp278-Glu323-Glu267* (*from adjacent subunit) (Fig. 3A through C). Mutation of Glu267 or Glu323 to alanine led to a complete loss in the enzyme activity and capsule degradation activity as analyzed by top agar and enzyme activity assays, while the mutation D278A showed a partial reduction in capsule degradation activity compared to the wild-type enzyme (Fig. 1A; Fig. S2A). Both mutants E267A and E323A were screened, but the latter was crystallized first, and it was utilized for co-crystal structure determination. The sugar-bound crystals were subsequently obtained by soaking the E323A crystals in the crystallization solution added with the hydrolyzed K2 CPS products for 18–24 h. The complex structure was solved at 1.58 Å resolution. Interestingly, the sugar binding changed the space group of the crystals from *P*2_1_ to *C*2, resulting in a single trimer of the enzyme in the asymmetric unit.

The product-bound structure revealed three equivalent intersubunit carbohydrate-binding grooves in a trimer; two were bound with a dodecasaccharide, while the third had only one tetrasaccharide (Fig. 3A). Surprisingly, adjacent to the residues Ala323 (mutated from Glu323) and Glu267* that served as a boundary (Fig. 3C), the bound dodecasaccharide was, indeed, divided into a tetrasaccharide and an octasaccharide, which corresponded to the one and two tetrasaccharide repeating units identified in the hydrolyzed products of K2 CPS, as mentioned above. This finding strongly supports Glu267 and Glu323 to be the catalytic residues, which allows for the numbering of the polysaccharide chain from −3 to +6 based on the direction from the non-reducing end to the reducing end (Fig. 3B and C). Furthermore, as judged by the clear electron density, we observed an *O*-acetylation at the C6-OH of +4 Man*p* (Fig. 3B).

The bound dodecasaccharide lies in an elongated intersubunit groove, with the orientation of its two-repeating-unit chain nearly parallel to the longitudinal axis of the β-helix and the single-repeating-unit perpendicular to the longitudinal axis (Fig. 3A and B), reminiscent of the intersubunit carbohydrate-binding site reported for Sf6 TSP and ФAB6 TSP (35, 42, 43). Specifically, the interaction of the single-repeating-unit with K2-2 was mediated by hydrogen bonds between the sugar and the residues Asp278, Glu323^WT^ (obtained by overlay with the wild-type structure), Pro325, Ser327, Arg331, Lys210*, Tyr212*, Asp264*, and Glu267* of the enzyme (Fig. 3C and D; Fig. S2B). Notably, the C6-carboxyl group at the branched glucuronic acid that connects to −3 Man*p* formed a salt bridge with the residue Arg331 and a hydrogen bond to Ser327 (Fig. 3D; Fig. S2B), suggesting a crucial role of this carboxyl group in the K2 CPS recognition by the enzyme. Moreover, the binding of the two-repeating-unit to the enzyme was stabilized by hydrogen bonds to the residues Gly383, Gln411, R413, Gly415, Asn541, Lys210*, Ser290*, Ser311*, Ser312*, and His340* (Fig. 3E; Fig. S2C), including the hydrogen bond from the acetyl group at the C6-OH of +4 Man*p* to the main chain of Gly383.

As the residues Lys210, Arg331, Gly383, and Arg413 of the enzyme are deeply involved in the sugar binding, we mutated these residues to alanine individually and investigated the effect of the mutations on the enzymatic activity of the enzyme. We found that, except for G383A, these mutations remarkably reduced the catalytic activity of the enzyme toward hydrolyzing K2 CPS (Fig. 3F). Moreover, as revealed by enzyme kinetic study, the mutation R331A lowered the binding affinity between K2 CPS and K2-2 by ~3.2-fold, resulting in a ~8-fold decrease in the catalytic efficiency (*k*_cat_/*K*_m_) of the enzyme (Fig. 3G). These findings demonstrate the crucial role of the salt bridge between Arg331 and the carboxyl group of the sugar in the K2-2 and K2 CPS interaction. In addition, to disrupt its interaction with the acetyl group, Gly383 was mutated to proline (Fig. S2D). The limited effects observed for G383P suggested that the acetyl group on +4 Man*p* does not contribute to the substrate specificity. This may reflect the random distribution of *O*-acetylation observed in the hydrolyzed products of K2 CPS.

Regarding the catalytic center, the bound products indicate a retaining mechanism of the enzyme. The distance between Glu323^WT^ and Glu267* is ~4.9 Å, in good agreement with the typical distance between the catalytic nucleophile and general acid/base in a retaining glycosidase, i.e., 4.5–5.5 Å (35). Furthermore, although the distances from Glu323^WT^ or Glu267* to the anomeric carbon of −1 Glc*p* are both ~3.0 Å (Fig. 3C), we noticed that Glu267* is situated on the opposite side of the C1-OH of −1 Glc*p*. This positioning suggests that Glu267 could be a potential candidate for the nucleophile during the catalysis of the enzyme.

### A single-residue mutation in the C-terminal domain disrupted the trimer formation

**Fig 4:**
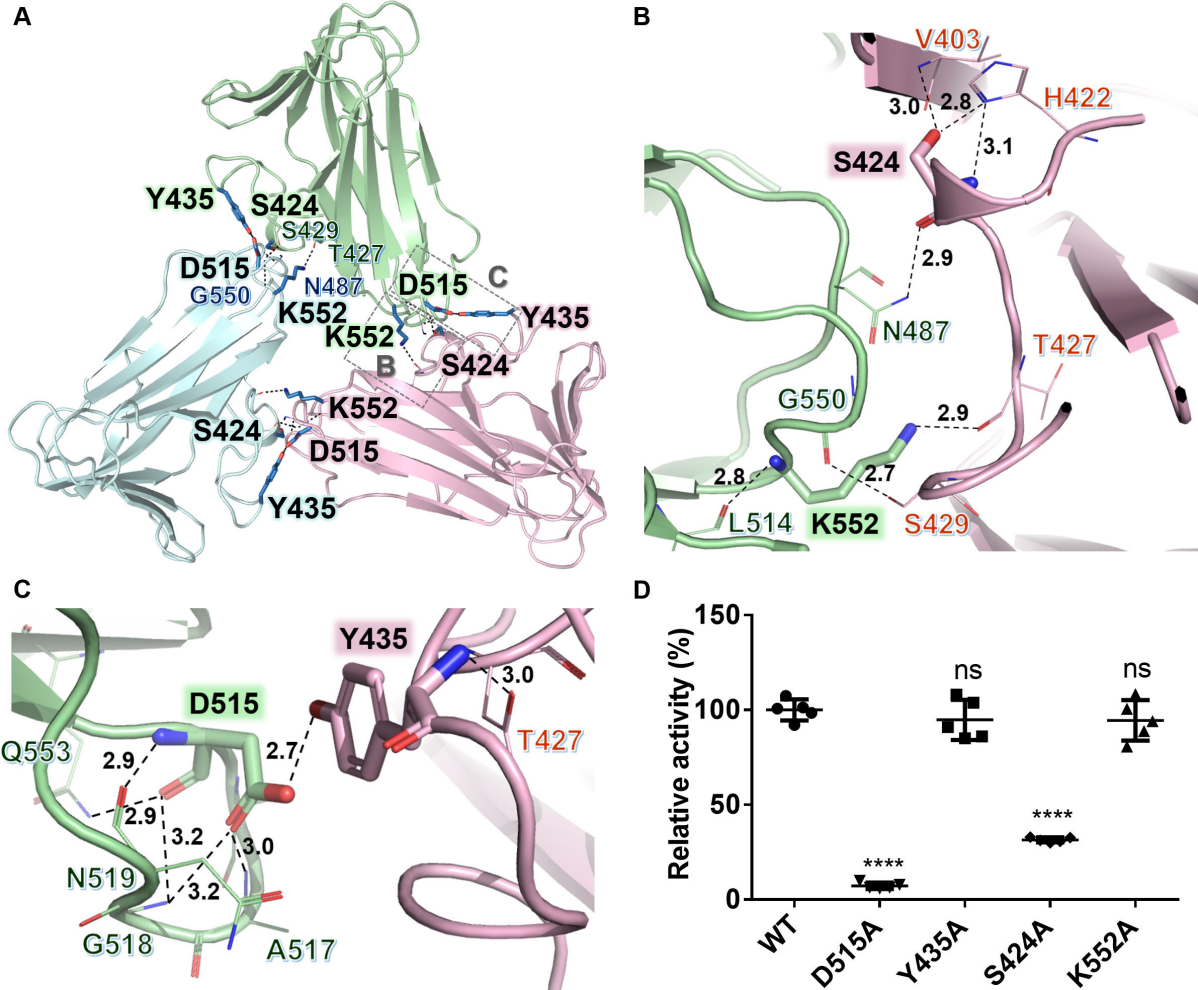
The inter-subunit interactions in the C-terminal domain of K2-2. (**A**) Residues situated in the dimer interfaces and involved in the intersubunit interactions of the C-terminal domain. (**B**) A close-up view of the hydrogen bond networks made by residues Asn487, Lys 514, Gly550, Lys552, Val403*, His422*, Ser424*, Thr427*, and Ser429* (*from adjacent subunit). (**C**) A close-up view of the hydrogen bond networks made by residues Asp515, Ala517, Gly518, Asn519, Gln553, Thr427*, and Tye435*. (**D**) Comparison of the CPS hydrolyzing activity of K2-2 mutants D515A, Y435A, S424A, and K552A with that of wild-type enzyme. The activities were measured under optimal pH and temperature conditions. Data are shown as means ± SD from five replicates. ****P < 0.0001, calculated from one-way ANOVA. ns, no statistical significance.

As indicated in the P22 TSP structure, the lethal mutations are mainly situated in the dimer interfaces of the C-terminal domain (30) despite the limited buried surface in this region. In the present study, analysis by PDBePISA (https://www.ebi.ac.uk/pdbe/pisa/) indicated that only ~5% of the solvent-exposed surface in the C-terminal domain of K2-2 is buried during trimer formation (Table S3). By examining the C-terminal structure of K2-2 (Fig. 4A), we found that the assembly of three β-sandwiches is primarily stabilized by the hydrogen bonds Asn487···Ser424*, Asp515···Tyr435*, Gly550···Ser429*, and Lys552···Thr427* at the dimer interfaces (Fig. 4B through C). Notably, Asp515 is further involved in an intra-chain hydrogen-bond network with Ala517, Gly518, Asn519, and Gln553 (Fig. 4C), and Ser424 also forms intra-chain hydrogen bonds with Val403 and His422 (Fig. 4B), suggesting that these two residues play important structural roles in both the monomer and trimer in the stabilization of the C-terminal domain of the enzyme.

To probe the contribution of these inter-chain and intra-chain hydrogen bonds to the trimer assembly and catalytic activity of the enzyme, the four residues Ser424, Tyr435, Asp515, and Lys552, which are deeply involved in the hydrogen-bond networks of dimer interfaces, were individually mutated to alanine. We found that the mutations D515A and S424A reduced the enzyme activity by ~92.5% and ~68.5%, respectively, while Y435A and K552A had nearly no effect on the enzyme activity (Fig. 4D). Furthermore, as analyzed by SEC-MALS, while the wild-type enzyme was absolutely a trimeric form in solution (Fig. S3A), the mutant S424A contained a minor fraction of monomer in addition to the main fraction of trimer (Fig. S3B). Surprisingly, D515A existed as a mixture of dimeric and monomeric forms in solution without the presence of trimer (Fig. S3C), indicating that the mutation D515A completely disrupted the trimer formation of the enzyme. By contrast, the other mutations described above did not affect the trimer formation of the enzyme (data not shown).

### An unusual tetrameric structure suggests the formation of a functional intersubunit catalytic center without trimerization

**Fig 5:**
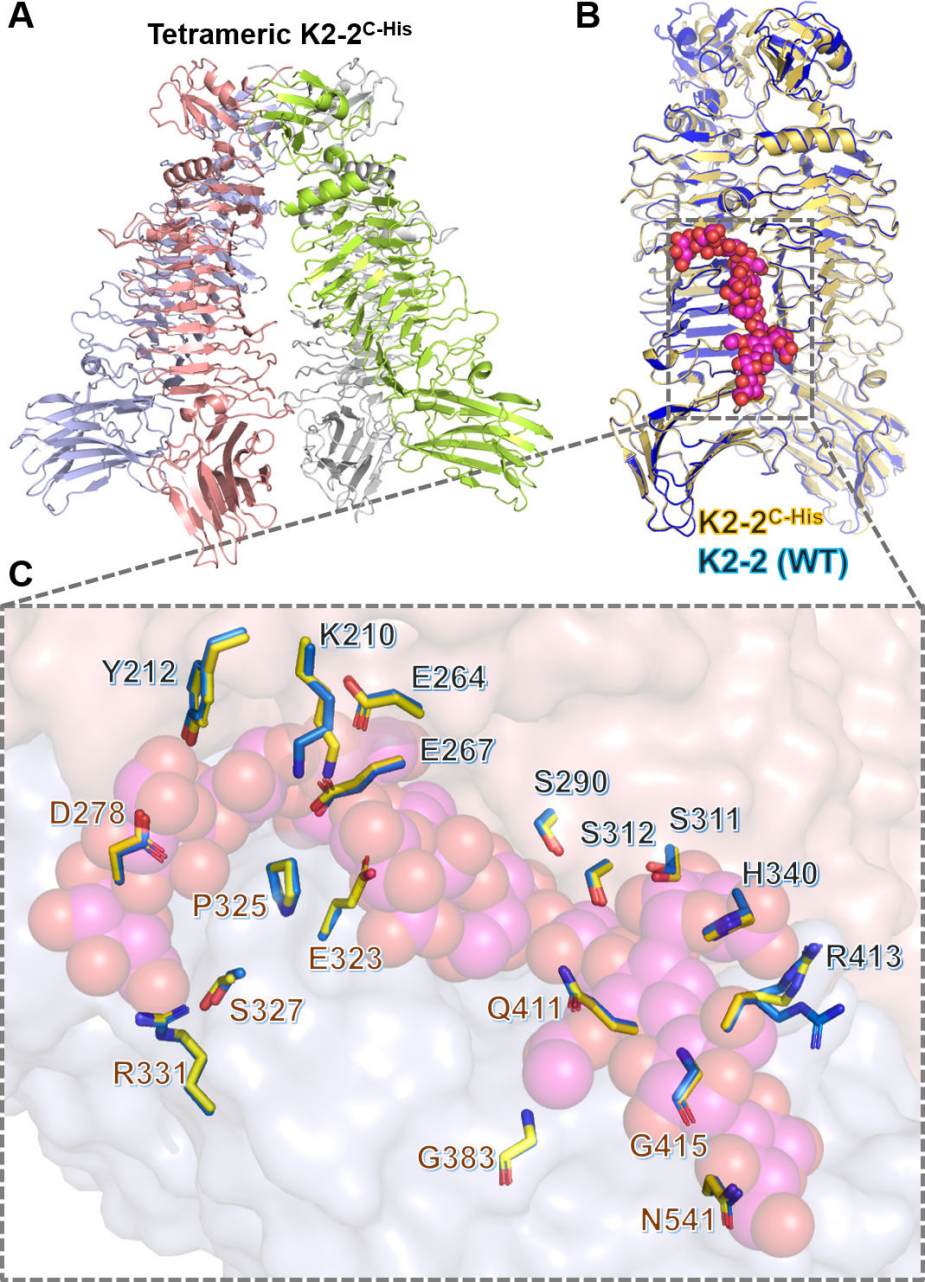
Crystal structure of the tetrameric form of K2-2^C-His^. (**A**) Overall structure of the tetrameric K2-2^C-His^ (PDB: 8IQE). The view is perpendicular to the tetramer axis with the N-terminal end on top. (**B**) Superimposition of the intersubunit carbohydrate-binding groove in the trimeric wild-type (WT) structure of K2-2 (dark blue) with that in the tetrameric structure of K2-2^C-His^ (pale yellow). The bound oligosaccharides in the latter structure are drawn as a sphere model in magenta.(**C**) A close-up view of the intersubunit carbohydrate-binding grooves. The residues involved in the binding to oligosaccharides in both structures are shown as a stick model, with color codes similar to those in panel B.

Before the trimeric wild-type structures were determined using the N-terminally His-tagged protein, the first crystals of K2-2 were, indeed, obtained by using a C-terminally His-tagged protein (termed K2-2^C-His^). The structure was solved at 2.17 Å resolution by the MAD phasing method under the space group of *P*6_3_ (Table S2). Unexpectedly, the solved structure revealed four protomers of the enzyme in an asymmetric unit, which formed a tetramer without the presence of a trimer. Specifically, two subunits are assembled into a dimer of a parallel left-handed helix, and two such dimers combine to form a right-handed superhelix (Fig. 5A). The two dimers superimpose well with each other, and also with any two adjacent subunits in the trimeric wild-type structure, presumably sharing a common interface. Further PISA analysis indicated that the interface strength of monomer-monomer in a dimer in the trimeric K2-2 was similar to that in the tetrameric K2-2^C-His^ (Table S4). This structure consolidates the importance of the C-terminal region in the trimer formation of the enzyme, reminiscent of previous studies on the lethal mutations of P22 TSP (30, 44), which indicated that the C-terminal domain of TSPs might function as a folding nucleus.

Regardless of the different overall organization, individual subunit in the tetrameric structure of K2-2^C-His^ exhibited a conformation nearly identical to that in the trimeric form. The intersubunit carbohydrate-binding groove could be overlaid well with that of the trimeric structure (Cα rmsd = 0.29 Å), including the residues involved in binding the sugar (Fig. 5B through C). Notably, as determined by top agar assay, this tetrameric form of K2-2^C-His^ possessed a significant capsule degradation activity towards the K2 *K. pneumoniae* (Fig. S4). SEC-MALS analysis showed that the majority of this K2-2^C-His^ protein existed as a tetrameric form in solution, but a minor fraction was still a trimer (Fig. S3D). Conceivably, although the electron density corresponding to the C-terminal His-tag was not visible in all subunits, the protruding His-tag might give rise to steric hindrance that interfered with the assembly of the enzyme into a trimer.

### Conclusion

A phage-derived polysaccharide depolymerase specific to the K2 CPS of *K. pneumoniae* was characterized. This depolymerase, named K2-2, broke the K2 CPS down to one and two tetrasaccharide repeating units. Nearly half of the products contained *O*-acetylation, which was thought to be crucial to the immunogenicity of CPS. The structure of the enzyme bound to the products revealed a trimeric superhelical architecture with intersubunit carbohydrate-binding grooves that accommodated three tetrasaccharide units of K2 CPS. The structures, combined with site-directed mutagenesis, enabled for the identification of the catalytic residues Glu267 and Glu323. Moreover, the salt bridge between Arg331 and the carboxyl group of K2 CPS is the key interaction in K2 CPS recognition by the enzyme, but *O*-acetylation on K2 CPS has no contribution to the substrate specificity.

The C-terminal domain of TSPs has long been believed as a crucial factor in the trimer formation of the proteins (30, 34, 45, 46). However, unlike several other TSPs that contain interdigitated or swapped elements following the central β-helix domain, the K2-2 structure described here shows a very limited buried surface in the C-terminal domain, which results in a negative free energy of dissociation (Δ*G*^diss^) when analyzed by PDBePISA. Thus, this structure allowed us to easily define the residues involved in key inter-chain interactions in the C-terminal domain, which enabled further investigation into their contribution to the trimer formation through mutagenesis experiments. Among the residues we identified, Ser424 and Asp515 are involved in both inter- and intra-chain hydrogen-bond networks, while Tyr435 and Lys552 only participate in inter-chain hydrogen bonds. Site-directed mutagenesis study here demonstrated that the single-residue mutation D515A is sufficient to nearly completely disrupt the trimer formation, while the mutation S424A led to a partial disruption in trimerization. Notably, despite the absence of trimer, the mixture of D515A, which consisted of the dimer and monomer plus a contaminated protein from *E. coli*, still showed ~7.5% catalytic activity compared to the wild-type enzyme. As the monomer does not have enzyme activity, the activity of the dimer of D515A was estimated to be ~48% of wild-type enzyme, indicating that the dimeric form retained the functional intersubunit catalytic center. By contrast, the mutations Y435A and K552A had nearly no effect to the trimerization, indicating that the loss of single inter-chain hydrogen bond is not sufficient to disrupt the trimer formation. It is also suggestive that the stability and integrity of local structure near the dimer interface in individual subunit are important as well to the trimerization of the enzyme.

Finally, the structural and functional characterization of the tetrameric form of K2-2 here demonstrates, for the first time in the field of TPSs, that a functional intersubunit catalytic center could be formed without the need for trimerization. Our results, thus, provide new insights into *K. pneumoniae* K2 capsule degradation by a polysaccharide depolymerase and improve our understanding of the assembly and catalysis of phage TSPs. They also provide cues for the development of glycoconjugate vaccines against *K. pneumoniae* infection.

## MATERIALS AND METHODS

### Protein expression and purification

A plasmid with the K2-2 gene included was kindly provided by Prof. Jin-Town Wang from the National Taiwan University Hospital (Taipei, Taiwan), and the DNA fragment encoding full-length K2-2 was amplified by polymerase chain reaction (PCR) (the primers given in Table S5). The amplified DNA fragment was then inserted into pET16b (Novagen) via *Nde*I and *Hind*III cloning sites, and pET21b (Novagen) via *Nco*I and *Xho*I cloning sites, resulting in expression constructs that produce the K2-2 with a N-terminal or a C-terminal His-tag, respectively. The constructs were then transformed into *E. coli* expression host BL21(DE3) (Novagen). The cell was grown in LB broth with carbenicillin at 37°C until an OD600 of 0.6–0.8 was reached and then induced with 1 mM IPTG at 16°C overnight. The cells were harvested by centrifugation, resuspended in buffer A (20 mM Tris-HCl, 0.5 M NaCl, pH 8.0) with 20 mM imidazole, passed through a Nanolyzer to lyse the cells, and then cleared by centrifugation at 20,000 × *g* at 4°C for 20 min. The supernatant was loaded onto a 5 mL Ni Sepharose 6 FF resin column (Cytiva) and washed with 20 mM and 50 mM imidazole in buffer A to remove unbound materials. The target protein was eluted with buffer A containing 0.2 M imidazole. The eluted protein was concentrated using a 30 kDa Amicon (Merck) and dialyzed against buffer B (20 mM Tris-HCl, 0.1 M NaCl, pH 8.0). Further purification was performed by using a Superdex 200 increase 10/300 Gl column (Cytiva) in the Protein Purification and Centrifuge Facility of the Institute of Biological Chemistry, Academia Sinica (Taipei, Taiwan). The target protein was eluted at ~12 mL.

### Site-directed mutagenesis

The expression constructs for K2-2 mutants (E267A, D278A, E323A, K210A, R331A, G383A, G383P, R413A, S424A, Y435A, D515A, and K552A) were generated based on the wild-type plasmid as template. Two pairs of primers (F1 and R1; F2 and R2) were used to amplify the plasmids through two independent reverse PCRs. All primers used in this process are listed in Table S5. The resulting PCR product 1 and 2 were mixed, digested with *Dpn*I, denatured at 95°C for 15 min, annealed at 65°C for 15 min, and renatured at 25°C for 15 min. The final product was then transformed into BL21(DE3), and the mutations were confirmed by DNA sequencing.

### Crystallization and data collection

The K2-2^C-His^ was crystallized at 15°C by mixing the protein solution (20 mg/mL in buffer B) with an equal volume of the reservoir solution (0.1 M Bicine pH 9.0, 10% PEG-4000, and 10% polyacrylic acid 2100 sodium salt) via the hanging-drop vapor diffusion method. The wild-type K2-2 was crystallized at 15°C by mixing the protein solution (15 mg/mL in buffer B) with an equal volume of the reservoir solution (0.1 M Bicine pH 9.0 and 22% PEG-6000) via the sitting-drop vapor diffusion method. To obtain the product-bound structure, the crystals of E323A-mutant K2-2 were soaked in reservoir solution containing 10 mM hydrolyzed products of K2 CPS for 18 h. Before flash-cooling in liquid nitrogen, all the crystals were briefly immersed in reservoir solution containing 10% (vol/vol) glycerol as cryoprotectant. The X-ray diffraction data were collected at the beamlines TLS 15A1 or TPS 05A of NSRRC (HsinChu, Taiwan). A two-wavelength MAD data at 2.55 Å resolution for crystals of Se-labeled K2-2^C-His^ was collected at the beamline 44XU of SPring-8 (Hyogo, Japan). All data were processed with the *HKL-2000* package (47).

### Structure determination and refinement

The crystal structure of K2-2^C-His^ was solved by the Se-MAD phasing method with the program *AutoSol* within *PHENIX* (48). Approximate 85% model was automatically traced into the Se-phased electron density map with the program *Buccaneer* (49), and the remainder was manually built with *Coot* (50). The resulting model was subjected to computational refinement with *Refmac5* (51, 52) and manual refinement with *Coot* until the *R*_work_/*R*_free_ values were converged. The crystal structures of the wild-type and the product-bound K2-2 were solved by the molecular-replacement phasing method with *Molrep* (53) based on the solved K2-2 structure above as search model (PDB: 8IQE). The resulting models were also subjected to computational and manual refinements. The bound oligosaccharides and well-ordered solvent molecules were located with *Coot*. The stereochemical quality of the refined models was checked with *MolProbity* (54). Final refinements were carried out with *PHENIX* and some statistics are listed in Table S2. Molecular figures were produced with *PyMOL* (Schrödinger, USA). The coordinates and structure factors have been deposited in the Protein Data Bank with accession codes 8IQ5, 8IQ9, and 8IQE.

### Extraction of K2 CPS

To prevent lipopolysaccharide contamination during extraction, we used the *K. pneumoniae* K2 (NTUH-A4528*ΔwbbO*) strain, which lacks the O-antigen in the lipopolysaccharide. The bacteria were collected after overnight cultivation in LB agar and suspended in distilled water. The suspension was heated at 100°C for 10 min, cooled down, and centrifuged at 20,000 × *g* for 30 min to remove the debris of the bacteria. The polysaccharide was precipitated from the supernatant using ice-cool acetone and centrifuged at 20,000 × *g* for 30 min to collect it. To digest the nucleic acid from the crude extraction, ribonuclease A (Roche) and deoxyribonuclease I (Roche) were added to the digestion buffer (20 mM Tris, 5 mM CaCl_2_, 5 mM MgCl_2_, pH 7.5) at 37°C for 4 h. Proteinase K was then added to overnight digest proteins. The enzymes were denatured by heating at 100°C for 30 min, and the supernatant was clarified by centrifugation and dialyzed against distilled water using an 8–10 kDa cutoff membrane. Before proceeding to the following steps, the crude polysaccharide was incubated with 0.1 M NaOH at 37°C for 4 h to obtain the deacetylated sample. CPS was precipitated overnight at 4°C using 75% ethanol and collected by centrifugation at 8,000 × *g* for 10 min. CPS was resuspended with 1.5 M NaCl and precipitated with 75% ethanol again. CPS was collected by centrifugation, suspended in distilled water, dialyzed with distilled water using a 1 kDa cutoff membrane, and lyophilized.

### Evaluation of enzymatic activity by top agar assay

The *K. pneumoniae* K2 (NTUH-A4528*ΔwbbO*) was cultivated overnight at 37°C. The culture was mixed with warm 0.7% agar and plated on the top layer of a 1.5% LB agar plate. Once the agar solidified, 2 µL of K2-2 or its mutants (with a concentration of 0.1 mg/mL) were dropped onto the plate and incubated at 37°C for 6 h. The appearance of translucent halos on the agar plate indicated the enzymatic activity, which can be quantified by measuring the dimensions of the halos.

### Analysis of hydrolyzed K2 CPS products by mass spectrometry and NMR

The K2-2-hydrolyzed products of K2 CPS were purified first by using an open column packed with Bio-Gel P-6 resin (Bio-Rad). Mass spectrometry (MS) analysis was carried out in a LTQ Orbitrap XL ETD mass spectrometer (Thermo Fisher Scientific, San Jose, CA) equipped with a standard ESI ion source. The data were collected in the range of 500–2,500 *m*/*z* at a flow rate of 50 µL/min in 80% ACN/H_2_O with 0.1% FA by the Ultimate 3000 RSLC system from Dionex (Dionex Corporation, Sunnyvale, CA). The data were collected and processed by the GRC Mass Core Facility of Genomics Research Center, Academia Sinica, (Taipei, Taiwan). Additionally, a 5 mg K2 oligosaccharide preparation was dissolved in 6,000 µL 99.95% D2O and analyzed using the AVANCE 500 MHz NMR spectrometer. The ^1^H chemical shift of the D2O at δH 4.70 ppm was used as the internal standard for the reference. All spectra were carried out using the standard pulse sequences provided by Bruker at 303 K and processed by the Bruker *TopSpin* software.

### Evaluation of CPS hydrolyzing activity

The CPS hydrolyzing activity of K2-2 toward K2 CPS was evaluated by quantifying the produced reducing ends in the products with the reagent 3,5-dinitrosalicylic acid (DNS) (55). Glucose was used as a standard to produce the calibration curve. The extraction of *K. pneumoniae* K2 (NTUH-A4528*ΔwbbO*) CPS substrate (5 mg/mL) was treated with K2-2 and incubated for the given periods. The reactions were stopped by adding three times the volume of DNS reagent and heating at 100°C for 10 min. Subsequently, the activity was evaluated by measuring the absorbance at 540 nm using UV–Vis spectrophotometer (Infinite M1000 PRO, Tecan). The optimal conditions for K2-2 were determined at a range of pH values and temperatures in CHC buffer (20 mM citrate/HEPES/CHES, 100 mM NaCl). The activity was initially calculated as specific activity in IU, which is defined as micromoles of product per minute per micromole of protein, and presented as relative activity (the percentage of the activity of the wild-type K2-2 enzyme) for easy comparison with the wild type.

The kinetic parameters of K2-2 and R331A mutant toward CPS substrate were obtained by fitting the initial reaction velocities at various substrate concentrations (which were varied from approximately 2–25 mg/mL) using the Michaelis–Menten equation in *GraphPad 6.0*. Each reaction was run five repetitions with eight substrate concentrations under optimal conditions.

### Analysis of protein assembly by SEC-MALS

The size exclusion chromatography-multi-angle static light scattering (SEC-MALS) analysis was conducted in the Biophysics Facility at the Institute of Biological Chemistry, Academia Sinica (Taipei, Taiwan). The SEC-MALS system used was a 1260 infinity quaternary LC system (Agilent), in combination with four online detectors: laser light scattering (miniDAWN TREOS, Wyatt), quasi-elastic light scattering (QELS, Wyatt), refractive index (Optilab T-rEX, Wyatt), and ultraviolet detection. A one hundred microgram protein sample containing 20 mM Tris (pH 8.0), 100 mM NaCl, and 0.02% NaN3 buffer was injected onto an ENrich SEC 650 column (Bio-Rad) and run continuously at a flow rate of 0.5 ml/min. Data were collected and analyzed by *ASTRA 6.17* (Wyatt Technology, USA) with the dn/dc set to 0.185 mL/g and poltted by *GraphPad Prism 9.1.2* (GraphPad softward, LLC).

## SUPPLEMENTAL MATERIAL

File S1 mbio.03519-23-s0001.docx NMR spectra of hydrolyzed K2 CPS product.

Supplemental figures and tables. mbio.03519-23-s0002.docx Fig. S1 to S4; Tables S1 to S5.

ASM does not own the copyrights to Supplemental Material that may be linked to, or accessed through, an article. The authors have granted ASM a non-exclusive, world-wide license to publish the Supplemental Material files. Please contact the corresponding author directly for reuse.
