# Supplementary material for: Klebsiella pneumoniae K2 capsular polysaccharide degradation by a bacteriophage depolymerase does not require trimer formation
Source: mBio. 2024 Feb 13;15(3):e03519-23. doi: 10.1128/mbio.03519-23 (PMC10936425; doi:10.1128/mbio.03519-23)
Supplement: File S1 — NMR spectra of hydrolyzed K2 CPS product. [file mbio.03519-23-s0001.docx]

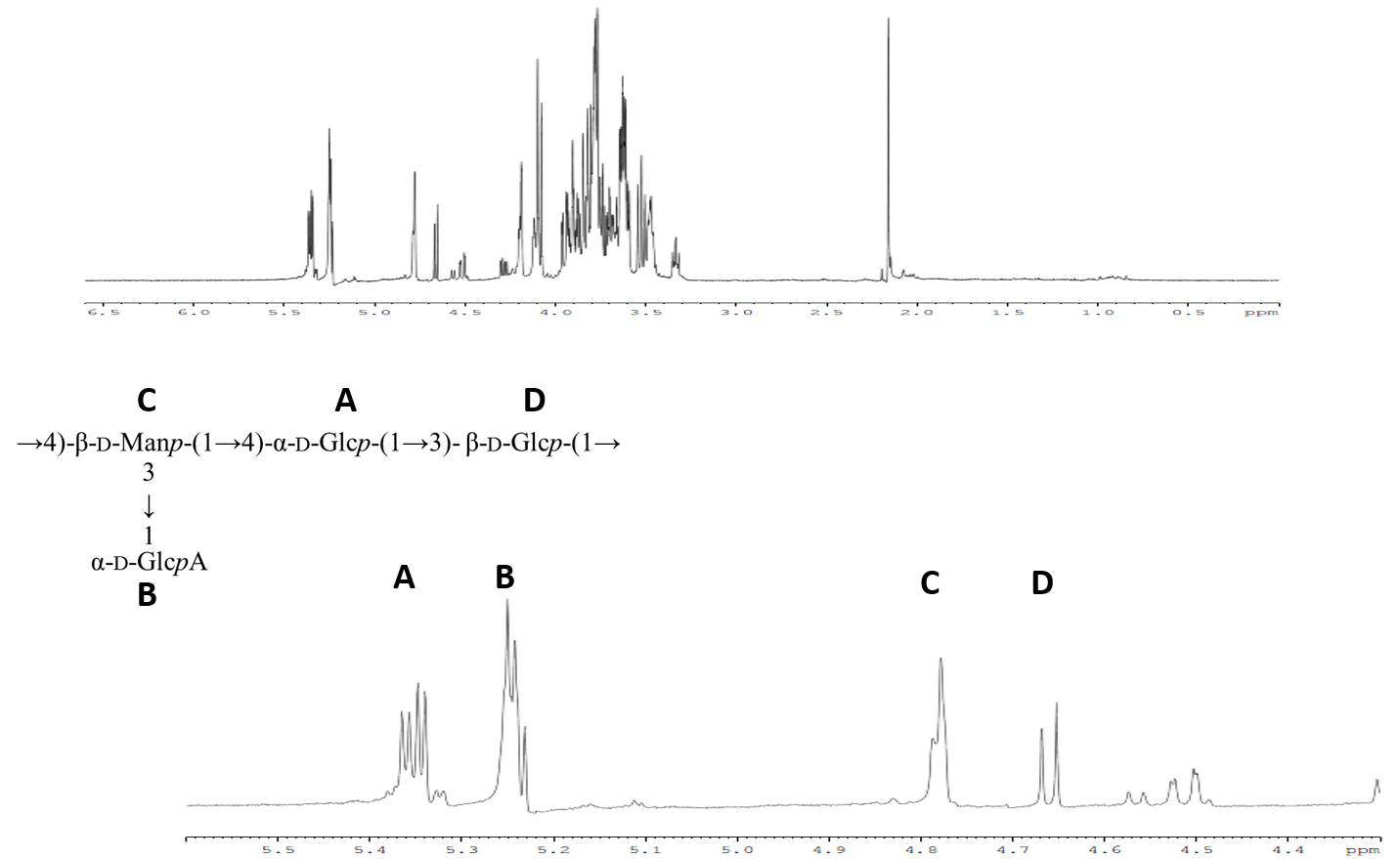


**Figure SF1. ^1^H NMR spectrum of K2-2 hydrolyzed CPS at 303 K.** (A), (B), (C), and (D) The chemical shift signals from α-D-Glucose, α-D-Glucuronic acid, β-D-Mannose, and β-D-Glucose, respectively.

**
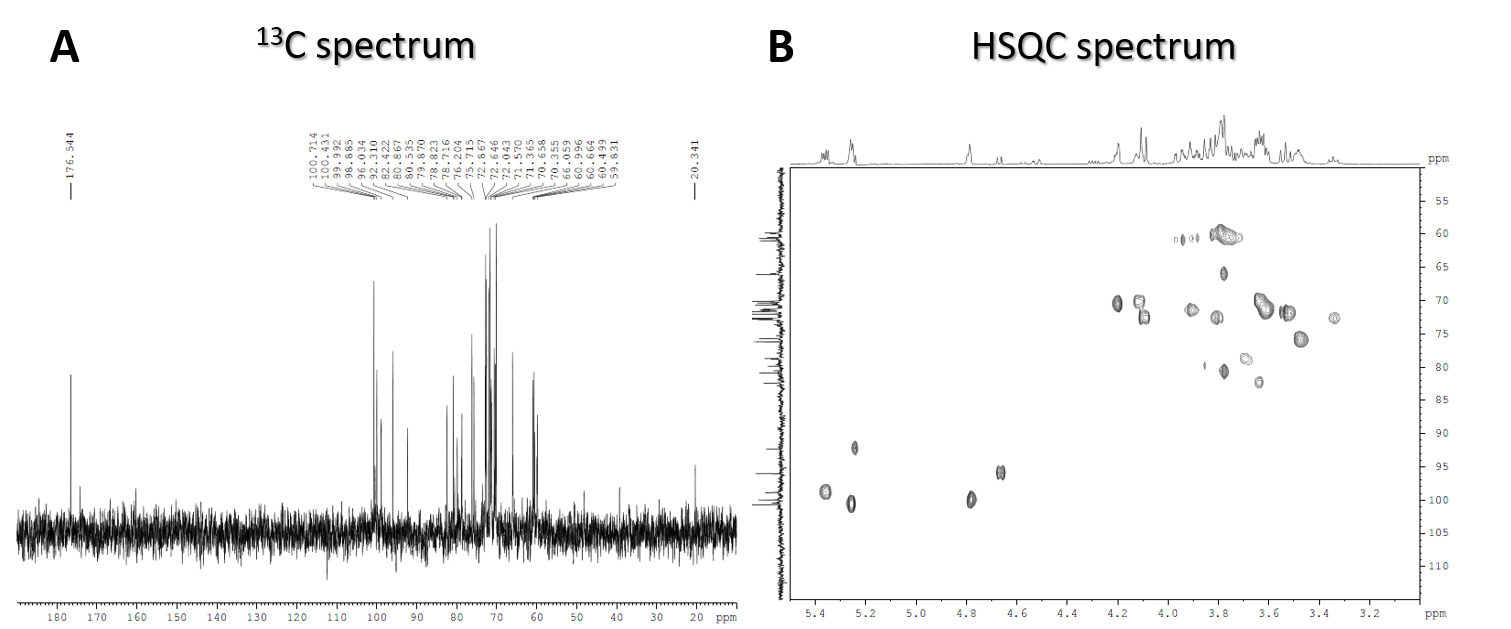
**

**Figure SF2. ^13^C (A) and HSQC spectrum (B) of K2-2 hydrolyzed CPS at 303 K.**

**
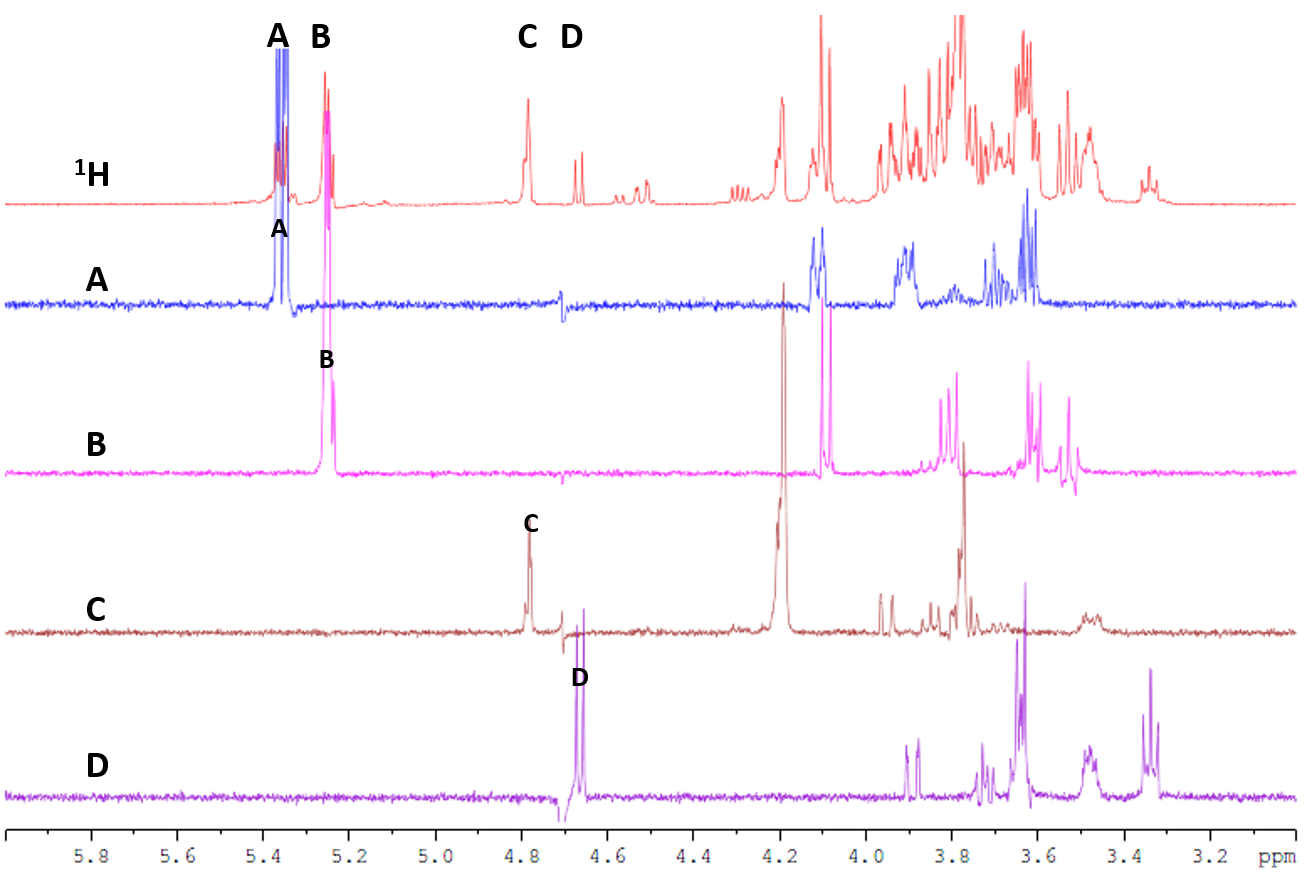
**

**Figure SF3. 1D TOCSY spectra of K2-2 hydrolyzed CPS at 303 K.**

**
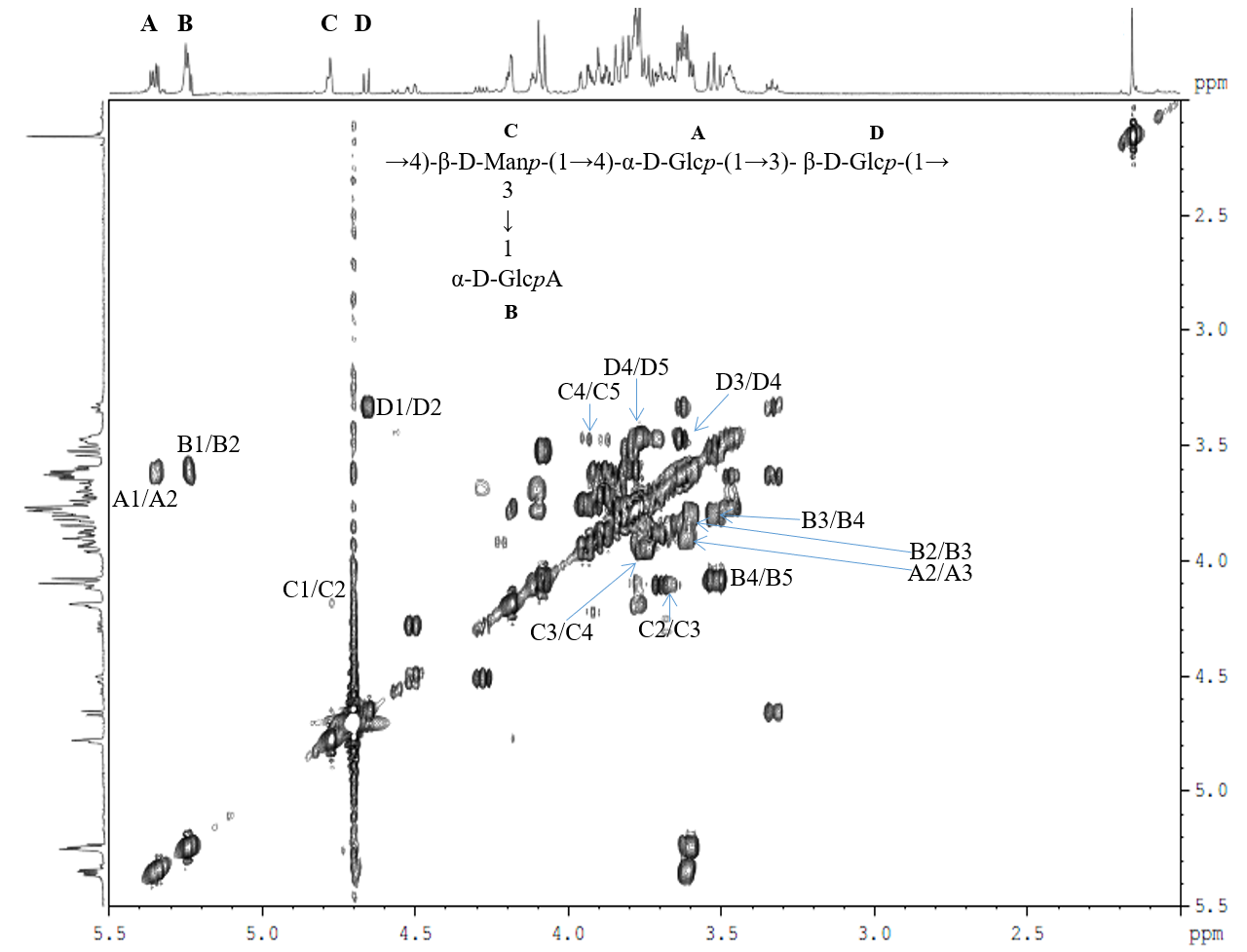
**

**Figure SF4. COSY spectrum of K2-2 hydrolyzed CPS at 303 K.**

**
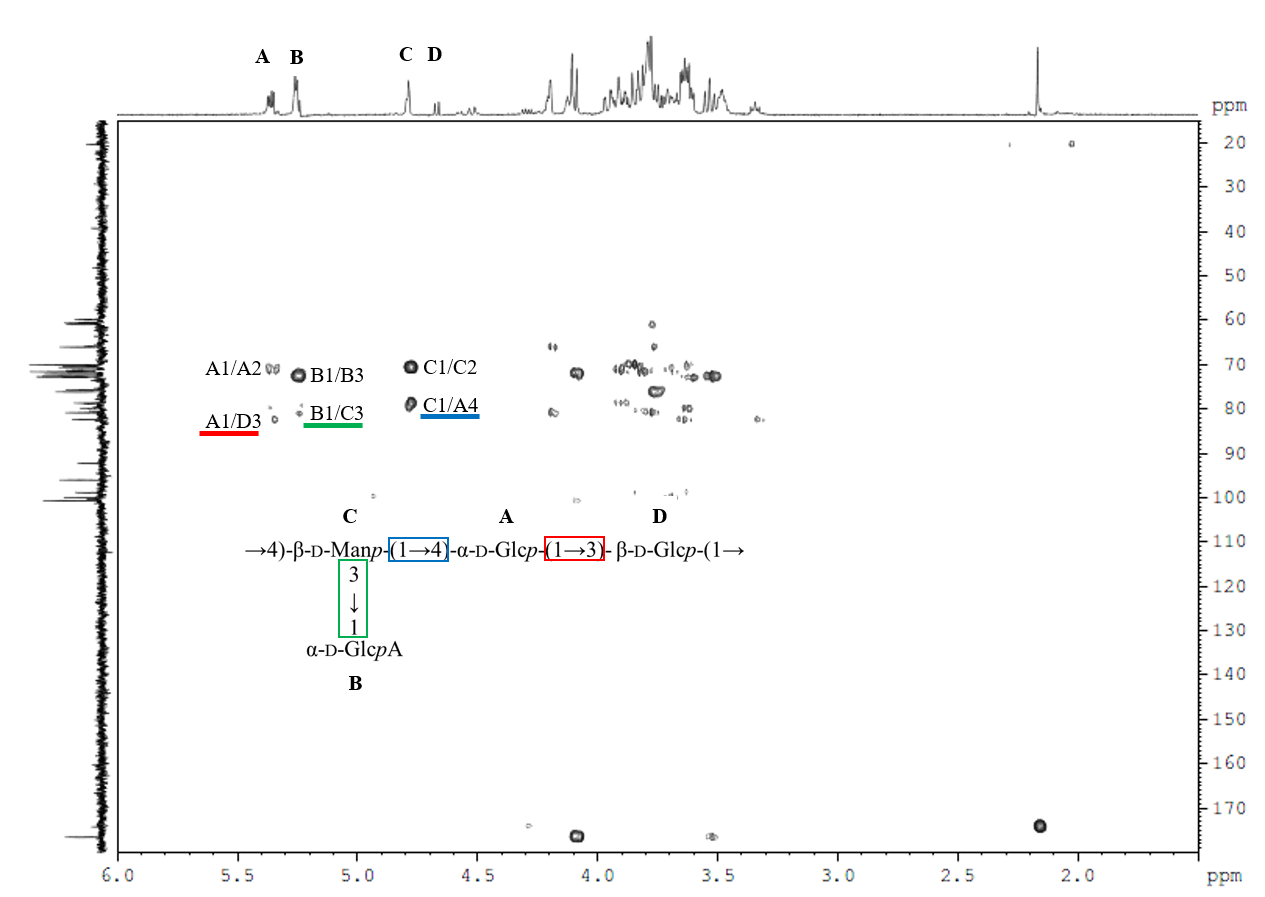
**

**Figure SF5. HMBC spectrum of K2-2 hydrolyzed CPS at 303 K.**

**
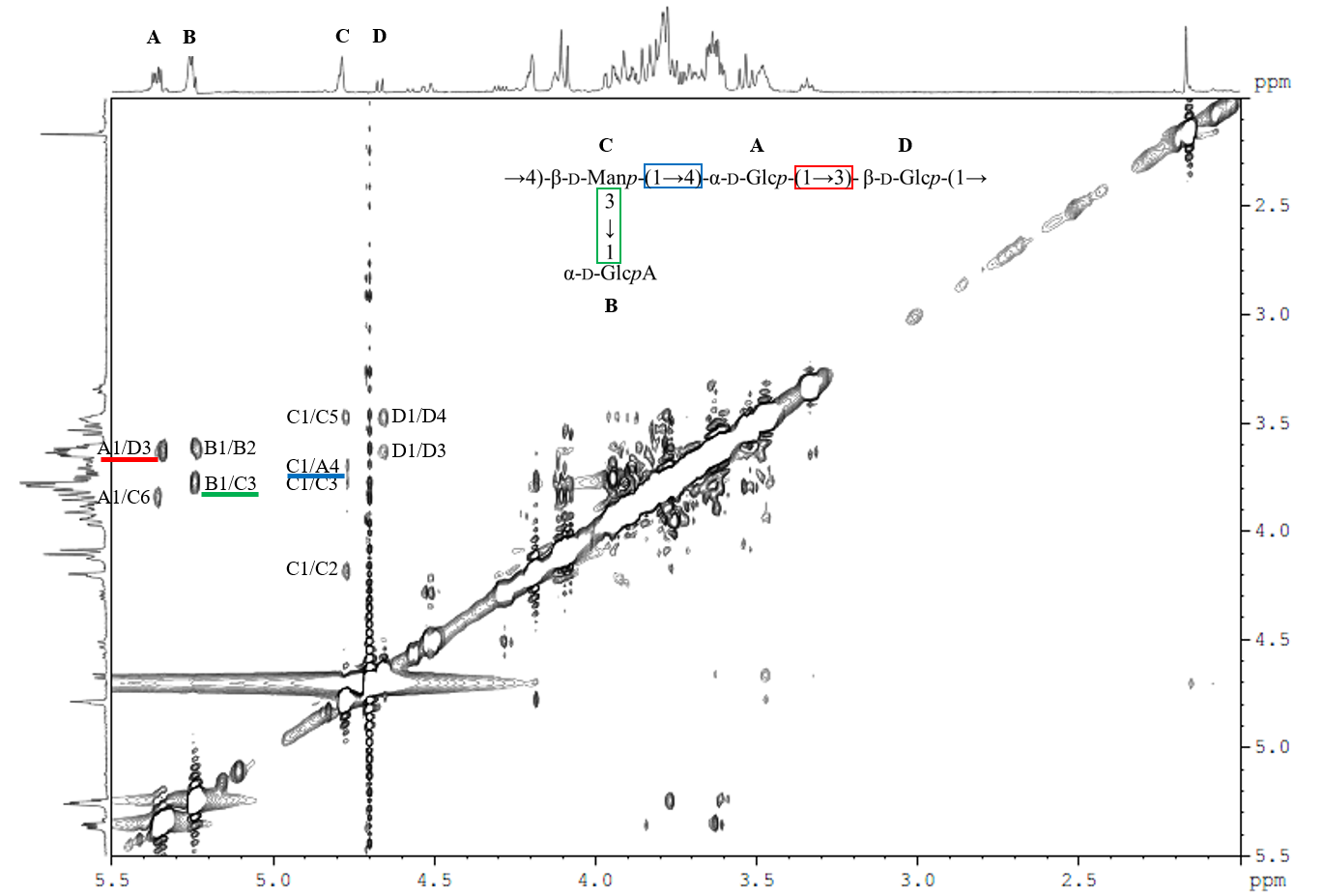
**

**Figure SF6. NOESY spectrum of K2-2 hydrolyzed CPS at 303 K.**

**
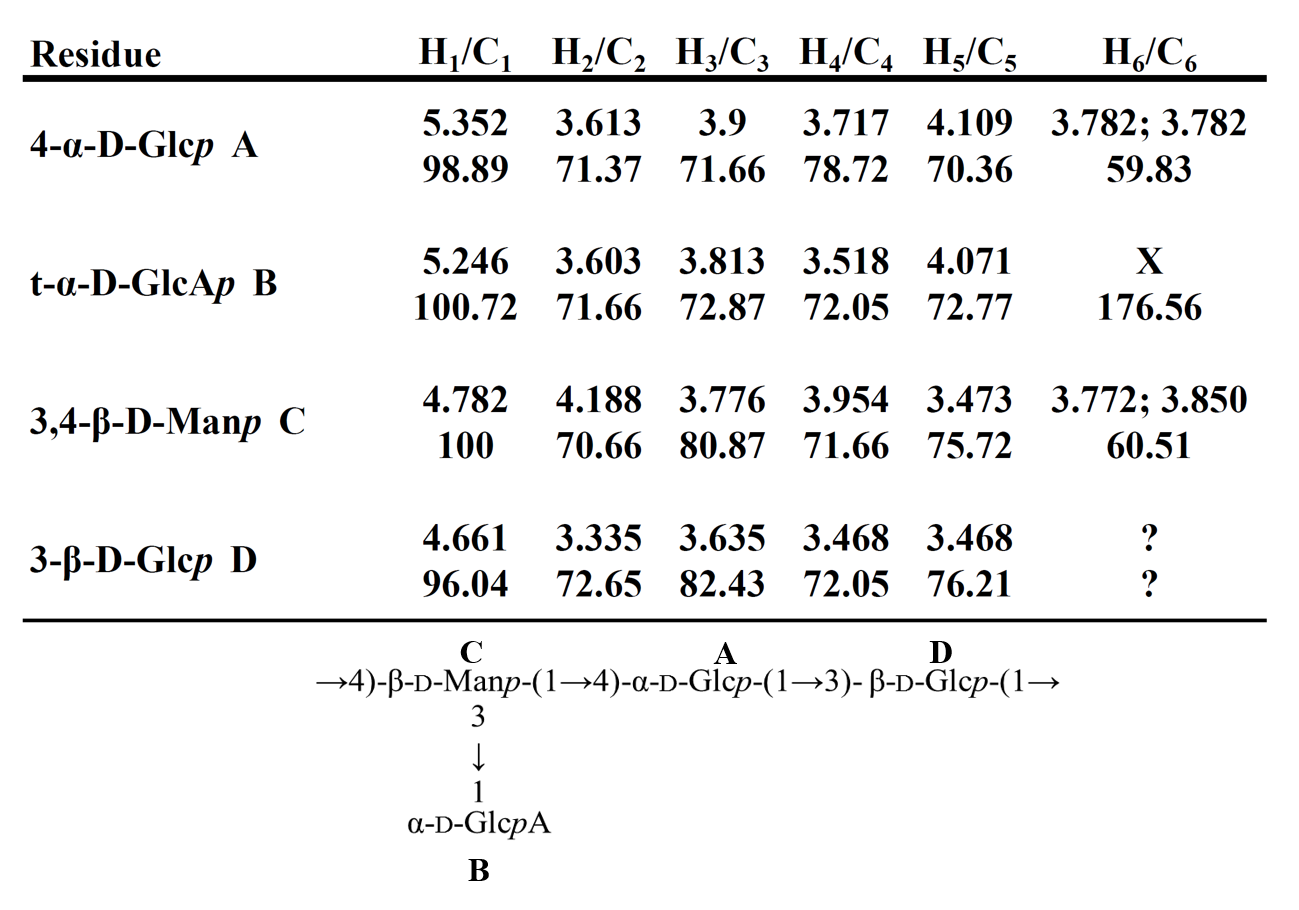
**

**Figure SF7. ^1^H and ^13^C chemical shifts (ppm) of K2-2 hydrolyzed CPS at 303K.**

**
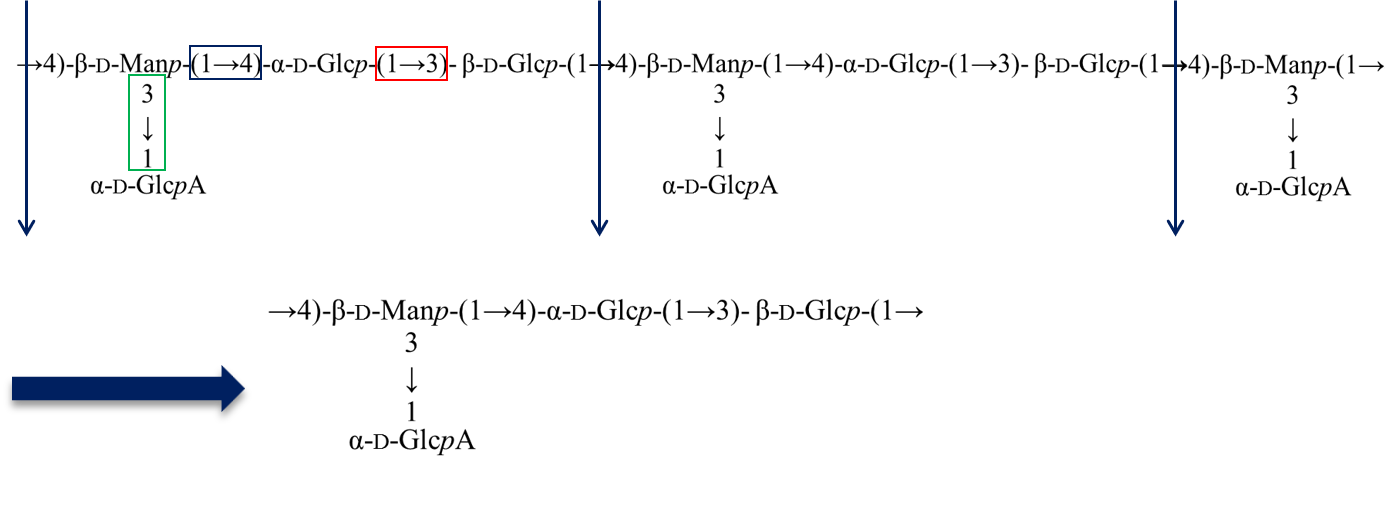
**

**Figure SF8. The cleavage sites on K2 CPS when treated with K2-2.** According to NOESY and HMBC spectra, the cleavage sites are located at the β-1,4 linkage between the glucose and mannose residues.
