# Supplementary material for: Klebsiella pneumoniae K2 capsular polysaccharide degradation by a bacteriophage depolymerase does not require trimer formation
Source: mBio. 2024 Feb 13;15(3):e03519-23. doi: 10.1128/mbio.03519-23 (PMC10936425; doi:10.1128/mbio.03519-23)
Supplement: Supplemental figures and tables. — Fig. S1 to S4; Tables S1 to S5. [file mbio.03519-23-s0002.docx]

**Table S1. Evaluation of capsule degradation activity of the bacteriophage encoding K2-2 against different capsule types of *K. pneumoniae***

| Phage specificity | |  |  |  |  |  |  |
| --- | --- | --- | --- | --- | --- | --- | --- |
| *K. pneumoniae*  Capsule type | Bacteriophage encoding K2-2 |  | K36 |  |  | K72 |  |
| K1 |  |  | K37 |  |  | K74 |  |
| K2 | ◎ |  | K38 |  |  | K79 |  |
| K3 |  |  | K39 |  |  | K80 |  |
| K4 |  |  | K40 |  |  | K81 |  |
| K5 |  |  | K41 |  |  | K82 |  |
| K6 |  |  | K42 |  |  | N1 |  |
| K7 |  |  | K43 |  |  | N2 |  |
| K8 |  |  | K44 |  |  | N3 |  |
| K9 |  |  | K45 |  |  |  |  |
| K10 |  |  | K46 |  |  |  |  |
| K11 |  |  | K47 |  |  |  |  |
| K12 |  |  | K48 |  |  |  |  |
| K13 | ◎ |  | K49 |  |  |  |  |
| K14 |  |  | K50 |  |  |  |  |
| K15 |  |  | K51 |  |  |  |  |
| K16 |  |  | K52 |  |  |  |  |
| K17 |  |  | K53 |  |  |  |  |
| K18 |  |  | K54 |  |  |  |  |
| K19 |  |  | K55 |  |  |  |  |
| K20 |  |  | K56 |  |  |  |  |
| K21 |  |  | K57 |  |  |  |  |
| K22 |  |  | K58 |  |  |  |  |
| K23 |  |  | K59 |  |  |  |  |
| K24 |  |  | K60 |  |  |  |  |
| K25 |  |  | K61 |  |  |  |  |
| K26 |  |  | K62 |  |  |  |  |
| K27 |  |  | K63 |  |  |  |  |
| K28 |  |  | K64 |  |  |  |  |
| K29 |  |  | K65 |  |  |  |  |
| K30 |  |  | K66 |  |  |  |  |
| K31 |  |  | K67 |  |  |  |  |
| K32 |  |  | K68 |  |  |  |  |
| K33 |  |  | K69 |  |  |  |  |
| K34 |  |  | K70 |  |  |  |  |
| K35 |  |  | K71 |  |  |  |  |

**Table S2. Data Collection and Refinement Statistics.**

|  | **MAD data** | | **Native** | | **Product-bound**  K2-2  (Trimer) |
| --- | --- | --- | --- | --- | --- |
|  | Se-labeled | | K2-2^C-His^  (Tetramer) | K2-2  (Trimer) |  |
|  | *High energy remote* | *Inflection* |  |  |  |
| **Data collection** | | | | | |
| Wavelength (Å) | 0.96372 | 0.9791 | 0.9 | 1.0 | 1.0 |
| Resolution (Å) | 30-2.55 | 30-2.55 | 30-2.17 | 30-1.38 | 30-1.58 |
| Space group | *P*6_3_ | *P*6_3_ | *P*6_3_ | *P*2_1_ | *C*2 |
| Unit cell dimensions | | | | | |
| *a, b, c* (Å) | 90.00, 90.00, 120.00 | 90.00, 90.00, 120.00 | 90.00, 90.00, 120.00 | 102.82, 82.84, 196.14 | 204.41, 86.36, 103.42 |
| *α, β, γ* (°) | 233.95, 233.95, 102.65 | 234.07, 234.07, 102.69 | 232.88, 232.88, 102.27 | 90.00, 98.37, 90.00 | 90.00, 110.21, 90.00 |
| Total observations | 796,087 | 790,251 | 828763 | 2568075 | 871912 |
| Unique reflections | 104,106 | 105,149 | 166451 (16573) | 651990 (63676) | 230579 (22985) |
| Multiplicity | 7.6 (7.6) | 7.5 (7.5) | 5.0 (4.9) | 4.0 (2.6) | 3.8 (3.7) |
| Completeness (%) | 100.0 (100.0) | 100.0 (100.0) | 100.0 (100.0) | 98.1 (96.2) | 100.0 (99.9) |
| *I*/σ(*I*) | 34.02 (3.19) | 31.29 (2.79) | 20.6 (2.0) | 24.1 (1.8) | 26.6 (2.4) |
| *R*_merge_ (%) | 13.1 (91.2) | 7.6 (85.7) | 7.3 (82.5) | 7.8 (56.0) | 5.5 (57.9) |
| **Refinement** | | | | | |
| Resolution (Å) |  |  | 29.56 - 2.17 (2.25 - 2.17) | 25.42 - 1.38 (1.43 - 1.38) | 26.95 - 1.58 (1.63 - 1.58) |
| Number of reflections |  |  | 158962 (10652) | 651918 (63255) | 230557 (22797) |
| *R_work_*/*R*_free_ |  |  | 0.1683/0.1978 | 0.1341/ 0.1578 | 0.1434/0.1729 |
| RMSD bond lengths (Å) |  |  | 0.0026 | 0.0123 | 0.0087 |
| RMSD bond angles (°) |  |  | 0.66 | 1.24 | 1.04 |
| B_ave_ (Å^2^) / Protein atoms |  |  | 38.8/16489 | 14.4/25533 | 21.8/12625 |
| B_ave_ (Å^2^) / Sugar atoms |  |  |  |  | 30.6/333 |
| B_ave_ (Å^2^) / Other atoms |  |  | 53.7/132 | 28.4/191 | 28.0/86 |
| B_ave_ (Å^2^) / Water molecules |  |  | 47.5/2081 | 32.2/5170 | 35.4/2240 |
| Ramachandran favored (%) |  |  | 97.05 | 96.50 | 96.32 |
| Ramachandran outliers (%) |  |  | 0.36 | 0.30 | 0.18 |
| Clashscore |  |  | 4.75 | 3.83 | 3.72 |
| MolProbity score |  |  | 1.45 | 1.40 | 1.41 |
| **PDB code** |  |  | **8IQE** | **8IQ5** | **8IQ9** |


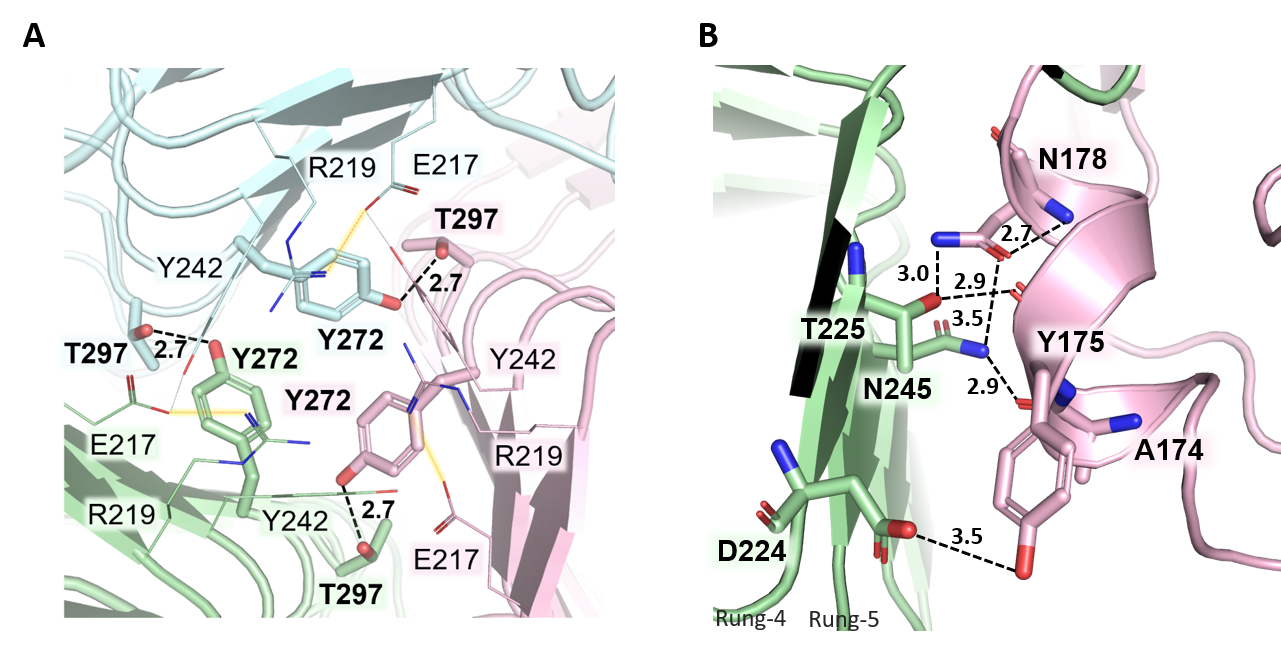


**Figure S1. Hydrogen-bond networks in the trimer interface and the protruding α-helix of the receptor binding domain.** (A) The inter-chain hydrogen-bond network formed by three pairs of Tyr272 and Thr297, each from a different subunit. (B) The protruding α-helix forms a hydrogen-bond network with the β2 strands of rung-4 and rung-5 from an adjacent β-helix.


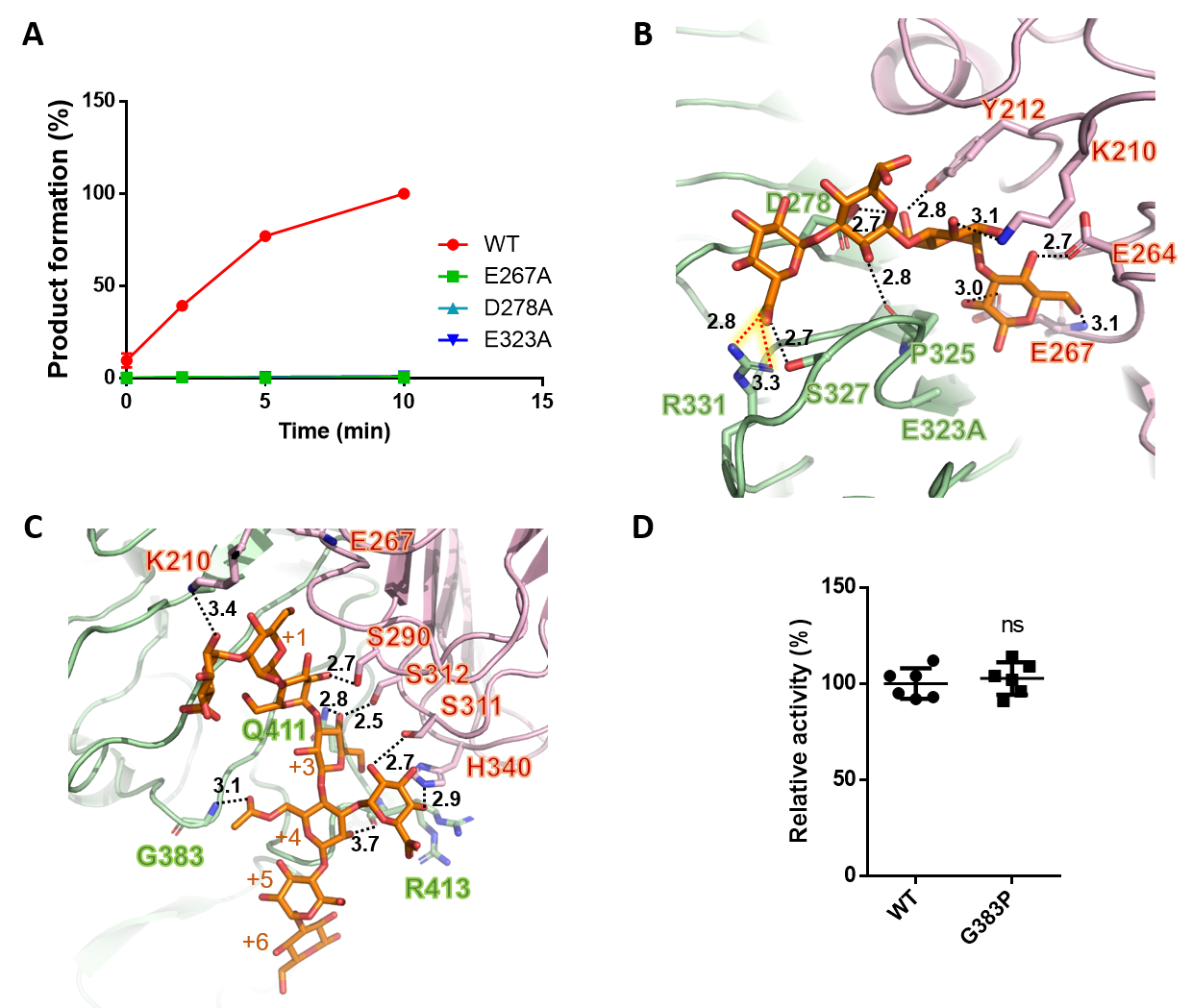


**Figure S2. CPS** **hydrolyzing activity of wild-type and mutant K2-2 and interaction between the enzyme and its hydrolyzing K2 CPS products.** (A) Evaluation of CPS hydrolyzing activity of the wild-type enzyme and the mutants E267A, D278A and E323A. (B) Interaction between the bound tetrasaccharide and K2-2. The dotted lines depict the possible hydrogen-bond interaction. (C) Interaction between the bound octasaccharide and K2-2. (D) CPS hydrolyzing activity of the mutant G383P and comparison with that of wild-type enzyme. The activities were measured under the optimal pH and temperature condition as described in the text. Data are shown as means ± SD from five replicates. ns: no statistical significance, calculated from Student's *t*-test.

**Table S3.** PISA analysis of the C-terminal domain

| C-terminal domain (residue numbers) | Surface area (Å^2^) | Buried area (Å^2^) | Buried area (%) | Δ*G*^diss^ (kcal/mol) |
| --- | --- | --- | --- | --- |
| 421 – 577 | 22384.8 | 1138.2 | 5.08 | -2.4 |


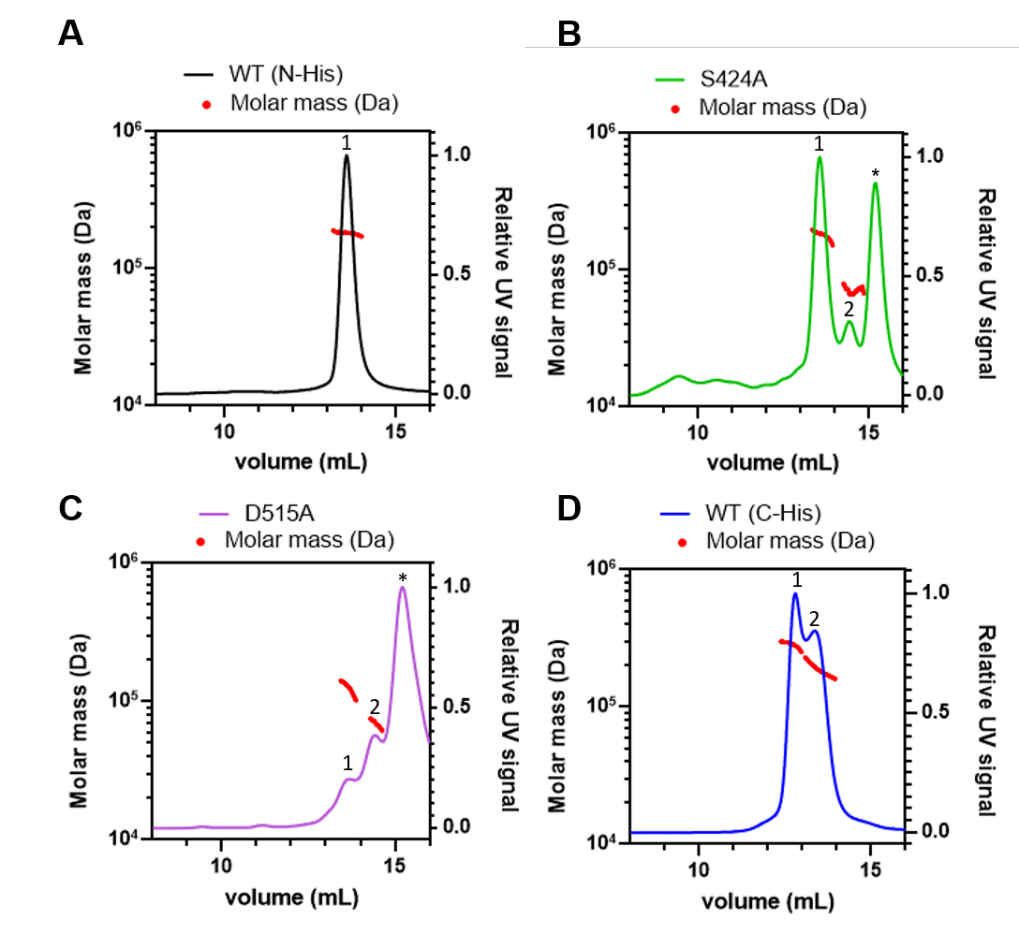


**Figure S3. SEC-MALS analysis of the assembly of wild-type and mutant K2-2 in solution.** (A) Wild-type K2-2. The molar mass of peak 1 was estimated to be 1.811 × 10^5^ (g/mole), corresponding to the trimeric form of the enzyme. (B) The mutant S424A. The molar mass of peak 1 and 2 were estimated to be 1.841 × 10^5^ (g/mole) and 6.994 × 10^4^ (g/mole), respectively, corresponding to the trimeric and monomeric form. The peak marked with * was found to be the contamination of trigger factor from *E. coli* cell during overexpression of the enzyme. (C) The mutant D515A. The molar mass of peak 1 and 2 were estimated to be 1.250 × 10^5^ (g/mole) and 6.897 × 10^4^ (g/mole), respectively, corresponding to the dimeric and monomeric form. (D) K2-2^C-His^. The molar mass of peak 1 and 2 were estimated to be 2.645 × 10^5^ (g/mole) and 1.931 × 10^5^ (g/mole), respectively, corresponding to the tetrameric and trimeric form.

**Table S4.** Comparison of buried surface in the dimer interfaces between the trimeric and tetrameric forms of K2-2 as analyzed by PIZA.

1. **K2-2 (Trimer)**

| K2-2  (Chains) | Average  Surface area (Å^2^) | Buried area (Å^2^) (Average) | Average  Buried area (Å^2^) |
| --- | --- | --- | --- |
| A – B  B – C  C – A | 21791.4 | 2757.5  2731.3  2710.4 | 2733.1 |

1. **K2-2^C-His^ (Tetramer)**

| K2-2^C-His^  (Chains) | Average  Surface area (Å^2^) | Buried area (Å^2^) (Average) | Average  Buried area (Å^2^) |
| --- | --- | --- | --- |
| A – B  C – D | 21423.5 | 2633.7  2487.3 | 2560.5 |


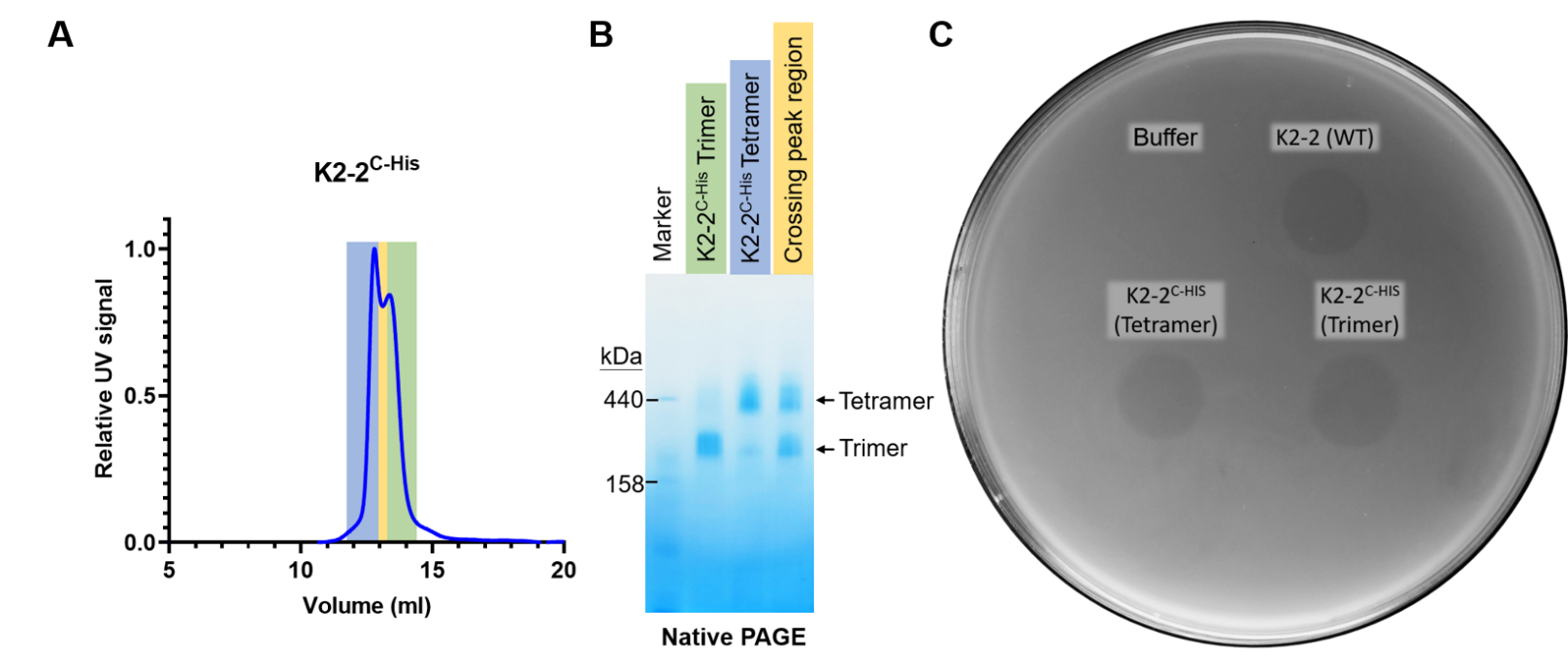


**Figure S4. Capsule degradation activity of K2-2^C-His^.** (A) The SEC analysis and (B) Native-PAGE analysis of K2-2^C-His^. Markers are Ferritin (440kDa) and Aldolase (158kDa) (Gel Filtration HMW Calibration Kit, Cytiva). (C) Evaluation of capsule degradation activity of the wild-type K2-2 and the tetrameric and trimeric forms of K2-2^C-His^ by top agar assay. The enzymes are spotted on plates pre-inoculated with the K2 *Klebsiella pneumoniae* NTUH-A4528*ΔwbbO*. Buffer (20 mM Tris-HCl and 0.1 M NaCl, pH 8.0) was used as the negative control.

**Table S5. Primers used for cloning**

| **To clone gene in expression vector** | **Primer Name** | **Sequence** |
| --- | --- | --- |
| **K2-2** | K2-2-F1 | 5’-TATGATGGCACTAGTAGATTTAGT-3’ |
|  | K2-2-F2 | 5’-TGATGGCACTAGTAGATT TAGT-3’ |
|  | K2-R1 | 5’-AGCTTCTTGACCACATTGCACAG-3’ |
|  | K2-2-R2 | 5’-TCTTGACCACATTGCACAG-3’ |
| **K2-2^C-His^** | K2-2-NdeI-F2 | 5’-GGGAATTCCATATGGCACTAGTAGAT-3’ |
|  | K2-2-XhoI-R2 | 5’-CCGCTCGAGCGGCTTGACCACATTGCA -3’ |
| **To mutate gene** | **Primer Name** | **Sequence** |
| **K210A** | K210A-F1 | 5’-CGGGCAGACCGAGGTTCG-3’ |
|  | K210A-F2 | 5’-ATCGGACGCGAGCTATAACGGG-3’ |
|  | K210A-R1 | 5’-CCCACTGTTTCCCTGCCA-3’ |
|  | K210A-R2 | 5’-TTATAGCTCGCGTCCGATCCCAC-3’ |
| **E267A** | E267A-F1 | 5’-CGTTCTACCACTGCCAGTTCTTCG-3’ |
|  | E267A-F2 | 5’-GACTCTGGGGCGATTCTGTCG-3’ |
|  | E267A-R1 | 5’-CGACAGAATCGCCCCAGAGTC-3’ |
|  | E267A-R2 | 5’-GTCATCTAGTCCGGCCGGTACATAC-3’ |
| **D278A** | D278A-F1 | 5’-AGTTCTTCGCTGGTGCAGGTAGTA-3’ |
|  | D278A-F2 | 5’-GTAGTAATATCCGATTATCTTGCTCCTCAT-3’ |
|  | D278A-R1 | 5’-CTGCACCAGCGAAGAACTGGC-3’ |
|  | D278A-R2 | 5’-GGCAGTGGTAGAACGACAGAATCT-3’ |
| **E323A** | E323A-F1 | 5’-TGGAAGTGCTAGTACCCGTAGATACGTTGAC-3’ |
|  | E323A-F2 | 5’-TGCAACTTCGCGAACCCTGGA-3’ |
|  | E323A-R1 | 5’-TCCAGGGTTCGCGAAGTTGCA-3’ |
|  | E323A-R2 | 5’-GCATCCGTTACAGGTTACTGTCGC-3’ |
| **R331A** | R331A-F1 | 5’-TTGACATTAGTGCTGGGCACACTA-3’ |
|  | R331A-F2 | 5’-CTAGTACCGCTAGATACGTTGACATT-3’ |
|  | R331A-R1 | 5’-CACTTCCAGGGTTCTCGAAGTT-3’ |
|  | R331A-R2 | 5’-CGTATCTAGCGGTACTAGCACTTCC-3’ |
| **G383A** | G383A-F1 | 5’-GCAGGAGCAGGAGCTCGG-3’ |
|  | G383A-F2 | 5’-TTACGGCGCGCACTACCAGCA-3’ |
|  | G383A-R1 | 5’-GGAGCAGTTACACCTACAAGGT-3’ |
|  | G383A-R2 | 5’-TGGTAGTGCGCGCCGTAAGGA-3’ |
| **G383P** | G383P-F1 | 5’-GCAGGAGCAGGAGCTCGG-3’ |
|  | G383P-F2 | 5’-TTACGGCCCTCACTACCAGCA-3’ |
|  | G383P-R1 | 5’-GGAGCAGTTACACCTACAAGGT-3’ |
|  | G383P-R2 | 5’-TGGTAGTGAGGGCCGTAAGGA-3’ |
| **R413A** | R413A-F1 | 5’-GGGTACGTGTCCTTTGCACTCC-3’ |
|  | R413A-F2 | 5’-GCAGCTAGCCAACGGGGCGG-3’ |
|  | R413A-R1 | 5’-AGCATAACCCCAGAGGTTGTGA-3’ |
|  | R413A-R2 | 5’-GCCCCGTTGGCTAGCTGCAGCATA-3’ |
| **S424A** | S424A-F1 | 5’-GTTCAGTAACTGGAATTTTGGTTATGGG-3’ |
|  | S424A-F2 | 5’-GCACTCCGCTCTTAGCACGTTCAGTA-3’ |
|  | S424A-R1 | 5’-AAAGGACACGTACCCGCCC-3’ |
|  | S424A-R2 | 5’-GTGCTAAGAGCGGAGTGCAAAGGA-3’ |
| **Y435A** | Y435A-F1 | 5’-GAATGCTTGGACGGTAGATAAGGG-3’ |
|  | Y435A-F2 | 5’-TTTTGGTGCTGGGAACCTGAATGC-3’ |
|  | Y435A-R1 | 5’-TTCCAGTTACTGAACGTGCTAAGA-3’ |
|  | Y435A-R2 | 5’-AGGTTCCCAGCACCAAAATTCCAGT-3’ |
| **D515A** | D515A-F1 | 5’-TAACAGTCTACCTGGTGGGGTAT-3’ |
|  | D515A-F2 | 5’-GTTCTTGGCAGCTGCCGGTAACAGT-3’ |
|  | D515A-R1 | 5’-CCAATAGATACCTGCCCCGC-3’ |
|  | D515AR2 | 5’-CCGGCAGCTGCCAAGAACCCAA-3’ |
| **K552A** | K552A-F1 | 5’-TGTGAACATTCAGACTGTGGCTGG-3’ |
|  | K552A-F2 | 5’-CGGCGCAGCGCAGGTTCGTG-3’ |
|  | K552A-R1 | 5’-ATTGGAACTTTGCCCCGCAAG-3’ |
|  | K552A-R2 | 5’-CGAACCTGCGCTGCGCCGATTG-3’ |
